# A root-specific NLR network mediates immune signaling of resistance genes against plant parasitic nematodes

**DOI:** 10.1093/plcell/koaf145

**Published:** 2025-06-24

**Authors:** Daniel Lüdke, Toshiyuki Sakai, Jiorgos Kourelis, AmirAli Toghani, Hiroaki Adachi, Andrés Posbeyikian, Raoul Frijters, Hsuan Pai, Adeline Harant, Juan Carlos Lopez-Agudelo, Bozeng Tang, Karin Ernst, Martin Ganal, Adriaan Verhage, Chih-Hang Wu, Sophien Kamoun

**Affiliations:** The Sainsbury Laboratory, University of East Anglia, Norwich Research Park, Norwich NR4 7UH, UK; Laboratory of Crop Evolution, Graduate School of Agriculture, Kyoto University, Mozume, Muko, Kyoto 617-0001, Japan; The Sainsbury Laboratory, University of East Anglia, Norwich Research Park, Norwich NR4 7UH, UK; The Sainsbury Laboratory, University of East Anglia, Norwich Research Park, Norwich NR4 7UH, UK; Laboratory of Crop Evolution, Graduate School of Agriculture, Kyoto University, Mozume, Muko, Kyoto 617-0001, Japan; JST-PRESTO, 4-1-8, Honcho, Kawaguchi, Saitama 332-0012, Japan; The Sainsbury Laboratory, University of East Anglia, Norwich Research Park, Norwich NR4 7UH, UK; Department of Biotechnology, Rijk Zwaan Breeding B.V., Fijnaart 4793, the Netherlands; The Sainsbury Laboratory, University of East Anglia, Norwich Research Park, Norwich NR4 7UH, UK; The Sainsbury Laboratory, University of East Anglia, Norwich Research Park, Norwich NR4 7UH, UK; Institute of Plant and Microbial Biology, Academia Sinica, Nankang, Taipei 11529, Taiwan; The Sainsbury Laboratory, University of East Anglia, Norwich Research Park, Norwich NR4 7UH, UK; Institute of Plant Molecular and Developmental Biology, Heinrich-Heine-University Düsseldorf, Düsseldorf 40225, Germany; SGS Institut Fresenius GmbH, TraitGenetics Section, Gatersleben 06466, Germany; Department of Phytopathology, Rijk Zwaan Breeding B.V., De Lier 2678, The Netherlands; Institute of Plant and Microbial Biology, Academia Sinica, Nankang, Taipei 11529, Taiwan; The Sainsbury Laboratory, University of East Anglia, Norwich Research Park, Norwich NR4 7UH, UK

## Abstract

Plant nucleotide-binding domain and leucine-rich repeat immune receptors (NLRs) confer disease resistance to many foliar and root parasites. However, the extent to which NLR-mediated immunity is differentially regulated between plant organs is poorly known. Here, we show that a large cluster of tomato (*Solanum lycopersicum*) genes, encoding the cyst and root-knot nematode disease resistance proteins Hero and MeR1 as well as the NLR helper NLR required for cell death 6 (NRC6), is nearly exclusively expressed in the roots. This root-specific gene cluster emerged in *Solanum* species about 21 million years ago through gene duplication of the ancient asterid NRC network. NLR sensors in this gene cluster function exclusively through NRC6 helpers to trigger hypersensitive cell death. These findings indicate that the NRC6 gene cluster has sub-functionalized from the larger NRC network to specialize in mediating resistance against root pathogens, including cyst and root-knot nematodes. We propose that some NLR gene clusters and networks may have evolved organ-specific gene expression as an adaptation to particular parasites and to reduce the risk of autoimmunity.

## Teaser

Tomato plants evolved a root-specific immune receptor network that mediates defense signaling against parasitic nematodes, providing insights into organ-specific plant immunity.

## Introduction

Plants possess immune receptors that can detect invading pathogens and trigger potent immune responses. Intracellular nucleotide-binding domain and leucine-rich repeat (NLR) immune receptors are the most abundant class of plant resistance genes and confer immunity against various pathogenic microbes and parasitic pests, including nematodes and insects (Jones et al. 2016; Kourelis and van der Hoorn 2018; Kourelis et al. 2021). NLRs recognize pathogen-secreted effector proteins and activate immune responses, typically culminating in a form of programmed cell death, known as hypersensitive response (Jones and Dangl 2006). Pathogen effector recognition by NLRs can occur directly, through interactions between NLRs and cognate effector proteins, or indirectly, through guarding of host effector targets by NLRs (van der Hoorn and Kamoun 2008). Despite significant advances in our understanding of NLR biology, fundamental questions about these receptors remain unanswered. For example, the degree to which NLR immunity is expressed differentially between plant organs is poorly understood (Millett et al. 2015; Deng et al. 2017; Barbey et al. 2019; Adachi et al. 2023). A meta-analysis of NLR gene expression patterns across plant species revealed distinct organ-specific expression profiles in monocot and Fabaceae plants, which primarily express NLR genes in roots, whereas Brassicaceae plants, such as Arabidopsis, show relatively higher NLR expression in shoots (Munch et al. 2018). Recently, a specific subset of NLR genes was shown to have a cell-specific, vasculature-enriched expression pattern, which is upregulated upon fungal infection (Tang et al. 2023). These findings suggest that plant cells in different organs, tissues and cell-types may have evolved to express distinct repertoires of NLR receptors as an adaptation to the parasites that attack specific host organs or cell-types (Munch et al. 2018; Adachi et al. 2019b; Tang et al. 2023). In this study, we address this question by demonstrating that an NLR gene network that confers resistance to plant-parasitic nematodes is specifically expressed in the roots of tomato (*Solanum lycopersicum*).

While a number of NLRs function independently, encompassing both effector sensing and immune signaling in a single unit (functional singletons), many NLRs establish pairs or larger networks with sensor-helper relationships (Wu et al. 2017; Adachi et al. 2019b). One well-characterized example of networked NLRs are helper NLRs required for cell death (NRCs) and their disease resistance sensors, which form complex receptor networks of coiled-coil (CC) type NLRs (CC-NLRs) in the lamiid clade of asterid plants (Gabriëls et al. 2007; Wu et al. 2017; Kourelis et al. 2022; Goh et al. 2024; Sakai et al. 2024). Similar to paired NLRs, NRC sensor NLRs have specialized in the detection of pathogen effectors and require NRC helpers for immune responses and the induction of a hypersensitive cell death (Kourelis and Adachi 2022; Contreras et al. 2023a). Upon pathogen activation, NRC sensors induce the oligomerization of NRC helpers through an activation-and-release model (Ahn et al. 2023; Contreras et al. 2023b). In the resting state, NRC helpers form an autoinhibited homodimer which subsequently assembles into a higher-order hexameric resistosome upon sensor activation, similar to the wheel-like pentameric resistosome structures of singleton CC-NLRs, such as HOPZ-ACTIVATED RESISTANCE 1 (ZAR1) and stem rust resistance 35 (Sr35) (Wang et al. 2019; Förderer et al. 2022; Ahn et al. 2023; Contreras et al. 2023b; Ma et al. 2024; Madhuprakash et al. 2024; Sakai et al. 2024; Selvaraj et al. 2024).

The NRC4 helper, ZAR1 and other CC-NLR resistosomes function as calcium ion (Ca^2+^) channels at the plasma membrane, an activity required for induction of the hypersensitive cell death (Wang et al. 2019; Bi et al. 2021; Jacob et al. 2021; Förderer et al. 2022; Liu et al. 2024). This activity depends on the very N-terminal α1 helix of the CC domain which assembles into a funnel-shaped structure that forms the resistosome membrane pore (Wang et al. 2019; Adachi et al. 2019a; Bi et al. 2021; Jacob et al. 2021; Förderer et al. 2022; Liu et al. 2024; Madhuprakash et al. 2024). The α1 helix is defined by the “MADA motif,” an ancient consensus sequence conserved in about 20% of CC-NLRs from dicot and monocot plants (Adachi et al. 2019a) and present also in bryophytes (denoted as MAEPL motif) (Chia et al. 2024). Remarkably, the MADA α1 helix motif has become non-functional in a large number of CC-NLRs, presumably because they have specialized into sensor NLR activities and rely on MADA-containing helpers for induction of a hypersensitive cell death (Adachi et al. 2019a, 2019b). The NRC network adheres to this evolutionary “use-it-or-lose-it” model: Whereas the NRC helpers carry the consensus MADA sequence, the massively expanded phylogenetic NRC sensor clade lacks sequences matching this motif (Adachi et al. 2019a, 2019b; Contreras et al. 2023a; Sakai et al. 2024). In fact, a major sub-clade of NRC sensors carries the ∼400 to 1,200 amino acid N-terminal Solanaceae Domain (SD) as an extension prior to the CC domain, a feature which would presumably preclude the formation of a ZAR1-type resistosome channel (Adachi et al. 2019c; Seong et al. 2020). The SD-CC-NLR sensor clade arose early in the NRC network evolution through the integration of sequences of unknown origin (Seong et al. 2020). This clade includes well-studied disease resistance proteins such as Mi-1.2, Rpi-blb2, Sw5b, R1 and Prf (Milligan et al. 1998; Brommonschenkel et al. 2000; Ballvora et al. 2002; van der Vossen et al. 2005; Mucyn et al. 2006), as well as the cyst nematode resistance protein Hero (Ellis and Maxon Smith 1971; Ernst et al. 2002) and the root-knot nematode resistance protein MeR1 (Frijters et al. 2021), both of which originate from the wild tomato species *Solanum pimpinellifolium*.

In plant genomes, NLR genes can be found as isolated genes (genetic singletons) or in gene clusters that vary from pairs to over a dozen genes (Michelmore and Meyers 1998; Meyers et al. 2003). In Arabidopsis, around half of the NLR genes occur in gene clusters that primarily arose from tandem gene duplication or unequal crossing-over events (Meyers et al. 2003; Van de Weyer et al. 2019; Lee and Chae 2020). Many pairs of sensor and helper NLRs are genetically clustered, often in head-to-head orientation, potentially to enable coordinated gene expression (Ashikawa et al. 2008; Nausaka et al. 2009; Okuyama et al. 2011; Césari et al. 2014; Huh et al. 2017; Białas et al. 2018). In contrast, functionally connected genes in the NRC network are not always genetically linked and can be dispersed across the genomes as in the case of tomato (Wu et al. 2017; Seong et al. 2020; Sakai et al. 2024).

Plant-parasitic nematodes, including the potato cyst nematodes *Globodera rostochiensis* and *Globodera pallida* and the root-knot nematode *Meloidogyne enterolobii*, pose substantial threats to agriculture by inflicting root damage and reducing crop yields. Management of nematode diseases is particularly challenging due to their rapid reproduction and adaptability to various environments (Siddique et al. 2022). Their impact extends beyond agriculture, as they can harm natural ecosystems by disrupting plant root systems, altering ecosystem composition and functioning (Gillet et al. 2017; Li et al. 2023). Consequently, understanding the biology and ecology of plant-parasitic nematode interactions is essential for effective disease management strategies in agricultural and natural contexts.

Several nematode resistance genes belong to the SD-CC-NLR sub-clade within the wider NRC superclade. One example is tomato Mi-1.2, an economically important root-knot nematode resistance gene which is encoded within an NLR gene cluster (Milligan et al. 1998). *Mi-1.2*-mediated immune responses depend on the helper NLR NRC4, despite their lack of genetic linkage (Wu et al. 2017). In contrast, the cyst and root-knot nematode resistance genes *Hero* and *MeR1* reside in orthologous gene clusters of genetically linked NLRs in *S. pimpinellifolium* (Ellis and Maxon Smith 1971; Ernst et al. 2002; Frijters et al. 2021). Given that Hero and MeR1 lack a MADA α1 helix sequence and carry an N-terminal SD domain, they are likely dependent on helper NLRs to function (Adachi et al. 2019a; Seong et al. 2020). However, the identity of their putative helper NLR(s) is yet to be determined, and it remains unknown whether the expression of these nematode resistance genes is root-specific.

In this study, we applied a computational pipeline to predict functional connections between NLR proteins based on the parameters of intergenic distance and phylogenomics. We hypothesized that mining genomes for gene clusters consisting of phylogenetically unrelated NLRs (mixed-clade gene clusters) would reveal previously unknown sensor-helper pairings. We applied this approach to tomato (*S. lycopersicum*), a species that carries ∼200 NLRs. Our analysis identified 7 mixed-clade NLR gene clusters in tomato, one of which carries orthologs of the nematode resistance genes *Hero* and *MeR1*, along with ∼10 paralogous NLRs we called Hero-cluster NLRs (HCNs) and 1 to 2 genes of the NRC helper NLR clade we called NRC6. Remarkably, HCNs, including the *Hero* and *MeR1* resistance genes, exclusively depend on NRC6 helpers to trigger a hypersensitive cell death. Furthermore, RNA-sequencing (RNA-seq) data from root and leaf tissues revealed nearly exclusive baseline expression of *HCNs* and *NRC6* in tomato roots. These results suggest that the *HCN* and *NRC6* genes may have evolved as a root-specific NRC sub-network that mediates nematode resistance in *Solanum* plants. Our findings offer insights into the evolution and function of NRC-network-mediated nematode resistance and further underscore the emergence of organ-specific NLR immunity in plants.

## Results

### Three of 7 tomato NLR-mixed phylogenetic clade gene clusters include NRC helpers

**Figure 1.**
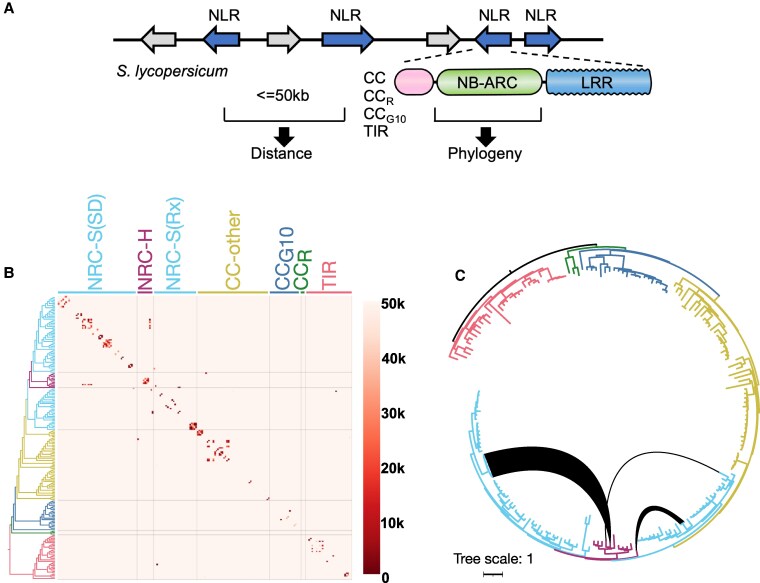
Tomato NLRs form clusters of phylogenetically related or unrelated NLR genes. **A)** Schematic representation of the computational pipeline employed for predicting clustering of sensor-helper NLRs. The genetic distances among tomato (*S. lycopersicum*) NLR genes and their phylogenetic relationships were assessed using a custom Python script available at https://github.com/slt666666/gene-cluster-matrix. **B)** NLR matrix integrating gene distance and phylogenetic relationships. The color scale bar on the right indicates distance between genes in kilo bases (kb). Genetically linked NLR genes (distance < 50 kb) are highlighted in the matrix, with colors representing gene distance and ordering based on phylogeny. The branches of major phylogenetic NLR clades (Wu et al. 2017; Kourelis et al. 2021) are color-coded, and NRC helpers (NRC-H) and NRC sensors (NRC-S) are depicted . The NRC sensor clade is further subdivided into Rx-type (Rx) or Solanaceous domain (SD)-containing NRC sensors. An interactive HTML version of the matrix containing detailed distance information on all NLR genes is provided as Supplementary Data S2. Coiled-Coil-type (CC), other CC-type (CC-other), G10-type CC (CC_G10_), RESISTANCE TO POWDERY MILDEW 8 (RPW8)-type (CC_R_), Toll/interleukin-1 receptor-type (TIR), Nucleotide-binding adaptor shared by APAF-1, certain R gene products, and CED-4 (NB-ARC), Leucine-rich repeat (LRR). **C)** Phylogenetic tree with black lines connecting NRC helper and NRC sensor NLRs encoded in gene clusters containing phylogenetically unrelated NLRs (mixed-clade gene clusters). Branches of the tree are color-coded according to B to indicate the phylogenetic clade. The scale bar represents evolutionary distance, measured as substitutions per site. Gene distance information, alignments, and phylogenetic tree files are available as Supplementary Data Set 1.

We used a computational pipeline to predict sensor-helper NLR gene clustering based on 2 parameters: genetic distance and phylogenomics (Fig. 1A; Sakai et al. 2024). Our hypothesis is that NLR gene clusters that consist of phylogenetically unrelated genes (mixed-clade clusters) include candidate sensor-helper relationships. To test this hypothesis, we analyzed the NLRome of tomato (*S. lycopersicum*). We used NLRtracker (Kourelis et al. 2021) to identify 197 NLRs from the genome annotations ITAG3.2 and NCBI RefSeq of tomato cv. Heinz 1706 (SL3.0) (Fig. 1A; Supplementary Data S1) and assigned them to the 7 major phylogenetic NLR groups based on prior classifications (Wu et al. 2017; Kourelis et al. 2021). The number of NLRs for these phylogenetic groups ranged from 3 (RESISTANCE TO POWDERY MILDEW 8 (RPW8)-type (CC_R_) clade) to 11, 24, 29, 31, 49 and 53 (NRC-H, G10-type CC (CC_G10_), NRC-S_Rx_, TIR, CC_other_, and NRC-S_SD_, respectively) (Fig. 1, B and C; Supplementary Data S1; Supplementary Data Set 1).

To determine the genomic distribution of the 197 tomato NLRs, we calculated the distance between each of the NLR-encoding genes (Fig. 1A, Supplementary Data Set 1) and created a genetic distance matrix in which the NLRs were grouped by phylogeny (Fig. 1, A and B; Supplementary Datas S2 and S3). Of the 197 NLRs, 107 (54%) were within 50 kb of another NLR gene and deemed to occur in 37 gene clusters consisting of 2 to 7 NLR genes per cluster (Fig. 1B; Supplementary Fig. S1A; Supplementary Data S3).

Next, we determined which NLR gene clusters contain mixed-clade genes. As evident from the genetic distance matrix, the great majority of NLR genes clustered with phylogenetically related NLRs, indicating the prevalence of tandem duplications during NLR gene expansion (Fig. 1B; Supplementary Datas S2 and S3). A total of 30 out of 37 NLR gene clusters included NLRs that belong to the same phylogenetic clade (Supplementary Fig. S1A; Supplementary Datas S2 and S3). However, we identified 7 mixed-clade NLR gene clusters (Fig. 1; Supplementary Fig. S1, Supplementary Datas S2 and S3). Notably, 3 of these gene clusters contain NRC helpers and NLRs from the NRC-dependent sensor clades, previously defined as NRC-S_Rx_ and NRC-S_SD_ (Fig. 1, B and C; Supplementary Fig. S1; Supplementary Datas S2 and S3; Wu et al. 2017; Kourelis et al. 2021). We considered NLRs encoded in these mixed-clade gene clusters as potential candidates for sensor-helper pairs or networks of the NRC phylogenetic clade.

### The cyst nematode resistance protein Hero is encoded in a large NRC mixed-clade gene cluster present across *Solanum* species

We noted that the largest mixed-clade NLR gene cluster identified contains homologs of the previously identified resistance gene *Hero* that functions against the potato cyst nematodes *G. rostochiensis* and *G. pallida* (Ellis and Maxon Smith 1971; Ernst et al. 2002), and an uncharacterized NRC helper clade protein (Fig. 1, B and C; Supplementary Fig. S1; Supplementary Datas S2 and S3). Hero belongs to the branch of the phylogenetic NRC superclade that includes NLRs with the SD N-terminal extension prior to the CC domain (NRC-S(SD) in Fig. 1B; Supplementary Datas S2 and S3; Supplementary Data Set 1). We reasoned that this gene cluster may consist of sensor-helper NLRs and further investigated this hypothesis.

**Figure 2.**
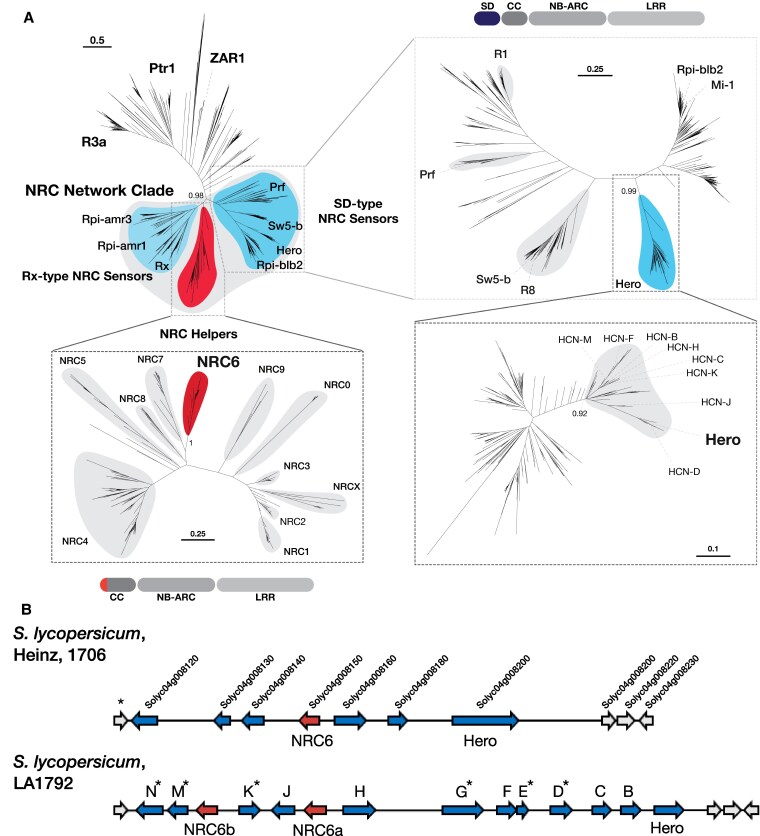
Hero and NRC6 form distinct phylogenetic subclades within the NRC helper and SD-type NRC sensor clades. **A)** The phylogenetic tree of NLRs was constructed based on the Nucleotide-binding adaptor shared by APAF-1, certain R gene products, and CED-4 (NB-ARC) domain from a large NLR sequence database (Supplementary Data Set 2). A total of 20.292 NLR sequences was aligned using MAFFT and the phylogeny was inferred using FastTree to determine the NRC network clades of NRC helpers, Rx-type NRC sensors and Solanaceous domain (SD)-type NRC sensors (top left). Sub-trees for the NRC helper (bottom left) and SD-type NRC sensor (top right) subclades were generated, containing well-defined phylogenetic clades including the Hero resistance protein and NRC6, respectively. The HCN clade is further divided into a tomato sub-clade (gray) which contains branches defined by proteins encoded in the NRC6 and HCN containing mixed-clade gene cluster (bottom right). The scale bar represents evolutionary distance, measured as substitutions per site. Coild-coil (CC), Leucine-rich repeat (LRR). **B)** Schematic depiction of the NRC6 and HCN encoding mixed-clade gene clusters of *S. lycopersicum* Heinz 1706 and LA1792, which contains an introgressed cluster from *S. pimpinellifolium* LA121 (Ellis and Maxon Smith 1971; Ernst et al. 2002). The HCNs encoded in this gene cluster define the branches of the tomato sub-clade shown in (A, bottom right). Gene IDs for the Heinz reference annotation are provided. Pseudogenes containing premature stop codons (Ernst et al. 2002) are indicated by an asterisk. SD-type NRC sensors are depicted in dark blue, NRC helpers in red, and non-NLRs in gray. Sequences, alignments, and phylogenetic tree file for A are available as Supplementary Data Set 2 or https://github.com/amiralito/Hero (Toghani et al. 2025). The LA1792 HCN sequences and cluster annotation is available under https://zenodo.org/records/10376142 (Lüdke et al. 2023).

The NRC helper clade protein encoded in this gene cluster is one of 11 NRC helper superclade proteins in tomato and was previously defined as NRC6 (Solyc04g008150.2; Wu et al. 2017). To determine the phylogenetic relationship of NRC6 and the genetically clustered Hero homologs, we constructed a phylogenetic tree with 20,292 NLR sequences extracted from a database derived from 124 plant genomes (Fig. 2A, top left; Supplementary Data S4). NRC6 belongs to a phylogenetic clade that includes the uncharacterized NRC family proteins NRC5, NRC7, and NRC8 and is more closely related to the functionally characterized NRC4 sub-clade than to the NRC1, NRC2, NRC3, and NRCX sub-clade (Fig. 2A, bottom left; Wu et al. 2017; Kourelis et al. 2021). Tomato NRC6 and the closely related NRC7 contain an atypical 12 amino acid N-terminal extension before their MADA motif, which scores lower compared to motifs of other functionally characterized NRC proteins when applying a Hidden-Markov Model for MADA motif detection (Supplementary Fig. S2; Adachi et al. 2019a). However, NRC6 is predicted to be a canonical helper NLR protein, containing an intact P-loop and MHD motif (Supplementary Fig. S2).

In the phylogenetic trees, the Hero resistance protein and other NLRs encoded in the *Hero* encoding gene cluster form a distinct phylogenetic clade (HCN clade) that is neighboring to the phylogenetic clade containing the nematode resistance protein Mi-1.2 (Milligan et al. 1998) and the potato late blight resistance protein Rpi-blb2 (van der Vossen et al. 2005) (Fig. 2A, top right). Mi-1.2, Rpi-blb2, and the HCNs all contain an N-terminal SD prior to the CC domain. Strikingly, the NRC6/HCN gene cluster is only detected in genomes of *Solanum* species, but is absent in the genomes of other Solanaceae, such as pepper (*Capsicum* spp.) or tobacco (*Nicotiana* spp.) (Supplementary Figure S3, Supplementary Data S4). We can therefore date the emergence of the NRC6/HCN gene cluster to around 17 to 21 million years ago (MYA) (Supplementary Fig. S3) based on divergence date estimates (Särkinen et al. 2013).

In the Heinz reference tomato genome assembly with the ITAG3.2 annotation, the NRC6/HCN gene cluster is annotated to contain 6 potential HCN genes of the phylogenetic NRC sensor clade and one NRC helper clade encoding gene, arranged in 2 blocks of tandemly repeated NLRs (Fig. 2). The *Hero* resistance gene was identified in an introgression line of cultivated tomato (*S*. *lycopersicum*, LA1792) and in the wild tomato species *S. pimpinellifolium* LA121 (Ellis and Maxon Smith 1971; Ernst et al. 2002). The introgressed wild tomato NLR gene cluster encodes 12 HCNs that are phylogenetically related to Hero, some of which appear to be pseudogenes (Fig. 2; Ernst et al. 2002). In addition, the gene cluster encodes for 2 NRC helper proteins, which phylogenetically cluster with NRC6 and were therefore termed NRC6a and NRC6b (Fig. 2). The comparison between the Heinz *S*. *lycopersicum* and the Hero *S. pimpinellifolium* NRC6/HCN gene cluster provides a glimpse of this cluster's sequence variation between *Solanum* species (Fig. 2B).

We compared the NRC6/HCN gene cluster extracted from chromosome-scale genome sequence assemblies of the *Solanum* species wild tomato (*S. pennelli*), potato (*S. tuberosum*), and American black nightshade (*S. americanum*), to the *S*. *lycopersicum* and the *S. pimpinellifolium* gene clusters. This revealed significant genomic rearrangements, presence/absence polymorphisms and duplications of the encoded NLR genes (Supplementary Fig. S4). In general, the extracted NRC6 sequences form a well-defined phylogenetic clade, whereas the HCNs appear more divergent, splitting into multiple subclades, as previously reported for NRC sensors of the Sd clade (Fig. 2A, bottom; Seong et al. 2020). However, the conservation of the NRC6/HCN gene cluster across multiple *Solanum* species (Supplementary Figs. S3 and S4) suggests that the gene cluster-encoded NRC6 and HCN proteins could be engaged in sensor-helper functional connections required for immune responses.

### HCNs require NRC6 helper NLRs to induce a hypersensitive cell death

**Figure 3.**
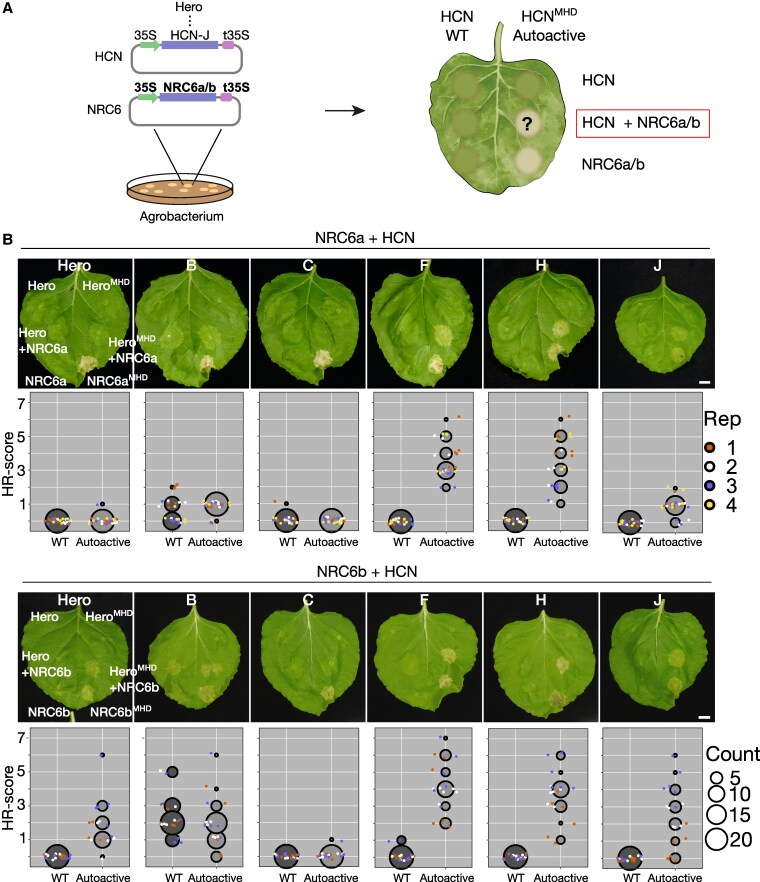
HCNs require NRC6 helper NLRs to trigger a hypersensitive cell death in *Nicotiana benthamiana*. **A)** The schematic representation illustrates the genetic complementation scheme employed throughout this experiment. Agrobacteria carrying NRC6a, NRC6b, or the specified HCN expression constructs were infiltrated into *N. benthamiana* leaves to express wildtype or autoactive HCNs and NRC6 helper NRCs (HCN^MHD^ or NRC6^MHD^, respectively) in the indicated combinations. **B)** Representative cell death phenotypes in *N. benthamiana* leaves induced by HCNs when co-expressed with NRC6a or NRC6b, photographed at 7 days post-infiltration (dpi) with agrobacteria. A p19 silencing construct was co-expressed in every infiltration. Cell death was visually scored and statistically analyzed. Data points are depicted as dots, with each biological replicate represented by a different color. The central circle for each cell death category proportionally represents the total number of data points for each treatment. Scoring is shown only for wildtype and autoactive HCNs, co-expressed with wildtype NRC6a or NRC6b helpers. The scale bar represents 1 cm. Each biological replicate (Rep) consists of 2 leaves from 3 different plants each. Complete quantification and statistical analysis are presented in Supplementary Fig. S5.

We hypothesized that Hero and other HCNs require their genetically clustered NRC6 for the induction of a hypersensitive cell death. To experimentally test this hypothesis, we used a genetic complementation assay based on transient expression by agroinfiltration in *Nicotiana benthamiana*, a species which lacks a functional NRC6 ortholog (Fig. 3; Supplementary Fig. S3; Adachi et al. 2019a). We first cloned Hero and 5 of its HCN paralogs along with NRC6a or NRC6b, extracted from the *S. pimpinellifolium* Hero gene cluster sequence of the introgression tomato line LA1792 (Ellis and Maxon Smith 1971; Ernst et al. 2002). We introduced a histidine (H) to alanine (A) mutation in the MHD motif of Hero and the other HCNs to generate autoactive mutants (van Ooijen et al. 2008) of the NLRs (referred to HCN^MHD^). In the co-expression assays, Hero caused macroscopic cell death in *N. benthamiana* leaves when co-expressed as MHD mutant with NRC6b, but not with NRC6a (Fig. 3B; Supplementary Figs. S5 and S6). In contrast, 4 of the 5 HCNs: HCN-B, HCN-F, HCN-H, and HCN-J, caused cell death when co-expressed as MHD mutants with either NRC6a or NRC6b (Fig. 3B; Supplementary Figs. S5 and S6). Among these HCNs, HCN-B also triggered cell death when co-expressed with NRC6a or NRC6b as both wildtype and MHD mutant variant (Fig. 3B). Of the 5 tested HCNs, HCN-C did not induce cell death either as wildtype or autoactive MHD mutant when co-expressed with NRC6a or NRC6b (Fig. 3B). However, in all cases, neither Hero nor the 4 functional HCNs caused cell death in the absence of the NRC6 proteins, indicating that these sensor-like autoactivated NLRs cannot signal for cell death without their helper NRC6 in *N. benthamiana* (Fig. 3; Supplementary Fig. S5). Expression of either NRC6a or NRC6b as autoactive MHD mutants induced a strong cell death, further outlining that NRC6 functions as a canonical helper (Fig. 3). We conclude that Hero and the HCNs are functionally connected with their genetically linked NRC6a and NRC6b helper NLRs and form a branch of the NRC network.

### NRC2, NRC3, and/or NRC4-dependent disease resistance proteins do not signal through NRC6

**Figure 4.**
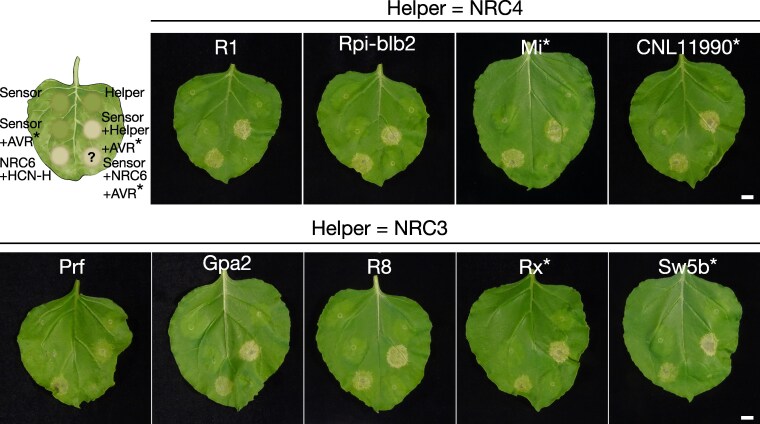
NRC2, NRC3 and/or NRC4-dependent disease resistance proteins do not signal through NRC6. The schematic representation illustrates the genetic complementation scheme employed throughout this experiment. Agrobacteria carrying expression constructs for sensors, helpers, or avirulence (AVR) effector genes were infiltrated in indicated combinations into the leaves of *N. benthamiana nrc2/3/4* mutant plants to trigger cell death. For Mi-1.2, CNL11990, Rx, and Sw5b, autoactive sensor mutants were expressed instead of a corresponding AVR effector gene (indicated by an asterisk). Tomato NRC3 or NRC4a was co-expressed as a positive control for complementation, respectively (Wu et al. 2017). HCN-H was used as a positive control for NRC6b-dependent cell death. Leaf images were photographed at 7 days post-infiltration (dpi) with Agrobacteria, a p19 gene silencing construct was co-expressed in each infiltration. The scale bar represents 1 cm. Quantification and statistical analysis are presented in Supplementary Fig. S7.

Our finding that the HCN and NRC6 gene cluster forms an NLR receptor sub-network within the much larger phylogenetic NRC superclade prompted us to determine the degree to which the network branches are functionally connected. To investigate this, we co-expressed with NRC6b the disease resistance proteins R1, Rpi-blb2, Mi-1.2, CNL11990, Pto, Gpa2, R8, Rx and Sw5b, all of which are known to be functionally dependent on NRC2, NRC3 and/or NRC4 (Wu et al. 2017). These NRC2/3/4-dependent sensors were either activated with their respective AVR effectors or expressed as autoactive mutants. In these assays, none of the 9 NRC2/3/4-dependent NLR proteins caused a visible cell death response in the presence of NRC6b, unlike the cell death response they triggered in the presence of the positive controls NRC3 or NRC4a (Fig. 4; Supplementary Fig. S7). We conclude that NRC6 forms a helper node in the NRC superclade network that is distinct from the previously characterized NRC2, NRC3 and NRC4 nodes. In addition, we also conclude that resistance to parasitic nematodes in the NRC network is mediated by multiple NRC helpers, given that, unlike Hero, the NRC2- and NRC3-dependent potato cyst nematode resistance protein Gpa2 and the NRC4-dependent root-knot nematode resistance protein Mi-1.2 function independently of NRC6 (Fig. 4).

### Hero and the HCNs do not signal through the other 9 tomato NRC helpers

**Figure 5.**
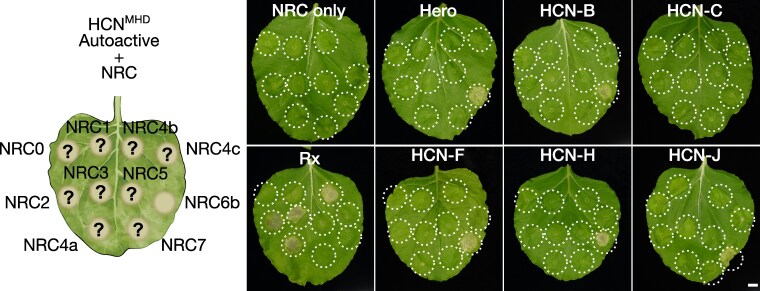
Hero and the HCNs do not signal through the other 9 tomato NRC helpers. The schematic representation illustrates the genetic complementation scheme employed throughout this experiment. Agrobacteria carrying autoactive HCNs (HCN^MHD^) and the indicated wildtype tomato NRC helper expression constructs were co-infiltrated into the leaves of *N. benthamiana nrc2/3/4* mutant plants. As a positive control, Rx was co-expressed with all tomato NRC helpers. Leaves were photographed at 7 days post-infiltration (dpi) with Agrobacteria, a p19 silencing construct was co-expressed for each infiltration. The scale bar represents 1 cm. Quantification and statistical analysis are presented in Supplementary Fig. S8.

Apart from NRC6a, NRC6b, and the NLR modulator NRCX (Adachi et al. 2023), there are 9 additional NRC helper proteins in the tomato NRC phylogenetic clade (Supplementary Fig. S2). To elucidate the relationship between NRC6 and other NRC clade proteins and to define the architecture of the NRC network, we co-expressed Hero and its 5 HCN paralogs with the other 9 tomato NRCs (Fig. 5). This enabled us to determine whether any of the HCN proteins could activate any of the other tomato NRC helpers. We co-expressed Hero and each autoactive HCN with each tomato NRC helper and used NRC6b as a positive control. We found that neither Hero, nor the other HCNs, could induce cell death through any other NRC proteins, except NRC6b (Fig. 5; Supplementary Figs. S8 and S9). As an additional positive control, we co-expressed the NRC sensor Rx with each of the tomato NRC helpers, confirming that Rx can activate NRC2, NRC3, variants of NRC4, as well as NRC1 from tomato, consistent with previous reports (Fig. 5; Supplementary Fig. S8; Gabriëls et al. 2007; Wu et al. 2017). It should be noted that although the tested NRC proteins expressed well in these experiments, we did not detect the accumulation of the NRC5 protein (Supplementary Fig. S9). In summary, Hero, HCNs and NRC6 form a specific NRC sub-network that is phylogenetically related, but distinct from the major NRC2, NRC3 and NRC4 network.

### The root-knot nematode resistance gene *MeR1* is a HCN that signals through NRC6

**Figure 6.**
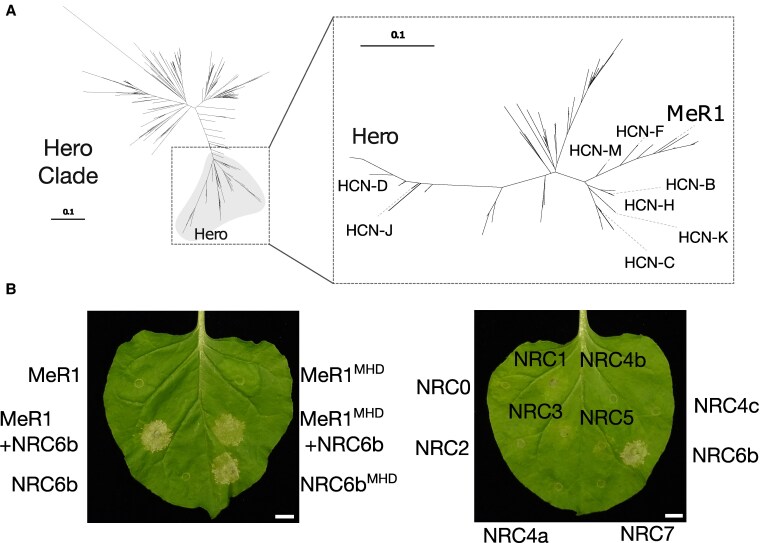
The root-knot nematode resistance gene MeR1 is a HCN that requires NRC6 to induce a hypersensitive cell death. **A)** A phylogenetic sub-tree was generated with MeR1 and tomato and potato NLRs defined as HCNs, from data presented in Fig. 2A. Re-alignment was performed using MAFFT, the phylogeny was inferred using FastTree. The scale bar represents evolutionary distance, measured as substitutions per site. **B)** Agrobacteria harboring wildtype or autoactive MeR1, or NRC helpers, were infiltrated in the indicated combinations into leaves of *N. benthamiana* wildtype (left panel) or *nrc2/3/4* mutant (right panel) plants for co-expression. Leaves were photographed at 7 days post-infiltration (dpi) with Agrobacteria, a p19 silencing construct was co-expressed for each infiltration. The scale bar represents 1 cm. Quantification and statistical analyses are presented in Supplementary Fig. S10. Sequences, alignments and the phylogenetic tree file are available as Supplementary Data Set 3.

In addition to the cyst nematode resistance protein Hero (Ellis and Maxon Smith 1971; Ernst et al. 2002), MeR1, a patented resistance protein against the root-knot nematode *M. enterolobii*, is also encoded within an orthologous HCN and NRC6 containing gene cluster in the wild tomato *S. pimpinellifolium* (Frijters et al. 2021). Based on phylogenetic analyses of HCN proteins, MeR1 is phylogenetically closest to HCN-F, raising the hypothesis of its specific dependence on NRC6 for cell death induction (Fig. 6A). To examine this hypothesis, we co-expressed MeR1 in *N. benthamiana* leaves, both in its wildtype and autoactive histidine to alanine (Mer1^MHD^) forms, with NRC6b or other tomato NRC helpers. Our results revealed that, similar to other HCNs, MeR1 exclusively signals through NRC6b, exhibiting no functional interaction with any of the other tomato NRC helpers we tested (Fig. 6B; Supplementary Fig. S10). In contrast, wildtype MeR1 or the autoactive MHD mutant did not induce cell death in *N. benthamiana* when expressed on their own (Fig. 6B; Supplementary Fig. S10). In these experiments, wildtype MeR1 displayed autoactivity when co-expressed with NRC6b, independently of the MHD mutation (Fig. 6B; Supplementary Fig. S10). We conclude that the NRC6/HCN gene cluster encodes resistance genes that function against 2 species of plant-parasitic nematodes, each with fundamentally different feeding strategies (Ernst et al. 2002; Frijters et al. 2021).

### HCNs and NRC6 helper genes are nearly exclusively expressed in tomato roots

**Figure 7.**
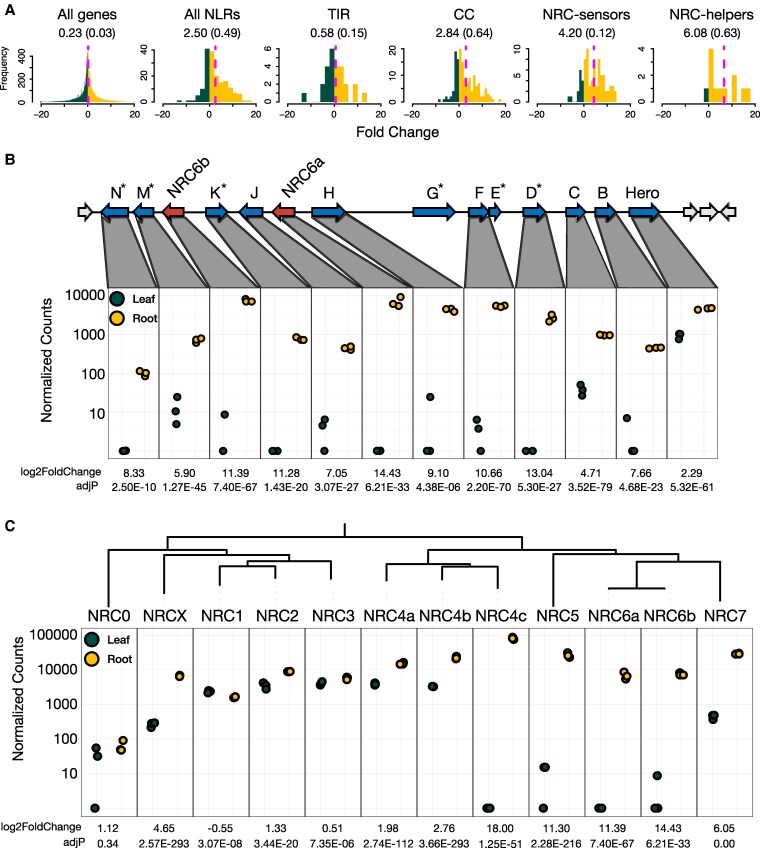
HCNs and NRC6 helper genes are nearly exclusively expressed in tomato roots. **A)** Histograms depicting the log2-fold change frequency, comparing the expression levels of all tomato genes, all NLRs, Coiled-Coil-type (CC), and Toll/interleukin-1 receptor-type (TIR) phylogenetic NLR subclades between roots and leaves of 2-week-old unchallenged tomato plants of the Hero introgression line LA1792 (Ellis and Maxon Smith 1971; Ernst et al. 2002). The dotted magenta line and numbers below labels indicate the mean log2-fold change, the skewness is indicated in brackets. Normalized counts for the expression of cluster-encoded HCN and NRC6 genes **B)**, and the expression of all tomato NRC clade genes **C)** in the roots and leaves of 2-week-old unchallenged LA1792 tomato plants. The log2-fold change and adjusted *P*-value are indicated for each gene. Pseudogenes are indicated by an asterisk. HCN-G and HCN-E pseudogenes exhibit negligible expression. Numbers for each gene are presented in Supplementary Table S1. The adjusted *P*-values (adjP) were calculated using DESeq2 with the Wald test and Benjamini-Hochberg correction for multiple testing. Complete data for log2-fold change values and normalized counts of all tomato genes and NLR subsets are available as Supplementary Datas S5 and S6.

Given that the potato cyst nematode *Globodera* species and the root-knot nematode *M. enterolobii* that are targeted by the NRC6/HCN resistance gene cluster are root pathogens, we investigated the organ-specific transcriptome profile of these NLR genes. To this end, we performed RNA-sequencing (RNA-seq) on leaf and root tissues of 2-week-old plants of the Hero introgression tomato line LA1792 (Ellis and Maxon Smith 1971; Ernst et al. 2002). The RNA-seq comparison between leaf and root samples provided consistent data across the 3 biological replicates (Supplementary Fig. S11). Notably, among the 175 NLR genes with detectable expression, 66% (56% having adj*P* < 0.05) exhibited a root-skewed expression pattern (mean log2FC = 2.05, skewness = 0.49). This contrasted with all expressed tomato genes, where 54% (36% with adj*P* < 0.05) of 28.185 expressed genes showed a root-skewed expression pattern (mean log2FC = 0.23, skewness = 0.03; Supplementary Data S5). This expression pattern of NLR genes can be primarily ascribed to CC-NLRs of the NRC superclade, which display high log2-fold changes for expression in roots (Fig. 7A).

Next, we examined the 12 genes in the NRC6/HCN gene cluster (Fig. 7B). All 10 expressed HCN genes displayed large log2-fold changes for root expression compared to leaves, ranging from 2.28 for Hero to 13.04 for HCN-D (Fig. 7B). NRC6a and NRC6b expression was also massively skewed towards roots with log2-fold changes of 14.43 and 11.39, respectively (Fig. 7B). Indeed, 9 of the 12 genes displayed hardly any RNA-seq signals in leaves, except for HCN-M, HCN-C, and Hero, which showed expression in leaves (Fig. 7B, Supplementary Data S6, Supplementary Table S1). Similar expression patterns can also be observed for the genes encoded in the Heinz tomato reference line (Tomato Genome Consortium 2012; Supplementary Fig. S12). To test the expression profile across different developmental stages, we used RT-PCRs on RNA extracted from leaf and root tissues of three-, four-, five-, and six-week-old plants of the Hero introgression tomato line LA1792, using primers specifically binding to *Hero*, *HCN-F*, *HCN-H*, *HCN-J*, *NRC6a*, or *NRC6b*. Consistent with our RNA-seq data, *Hero* displayed low expression in leaves and high expression in roots, while *HCN-F*, *HCN-H*, *HCN-J*, *NRC6a*, and *NRC6b* are exclusively expressed in roots across all developmental stages tested (Supplementary Fig. S13). Given that all HCNs require NRC6 as a helper for cell death induction, this suggests that the NRC6/HCN cluster genes act primarily in roots.

Since we could not detect the expression of *NRC6* in leaf tissues, we tested if expression of the autoactive HCNs could induce cell death in tomato leaves, which encode for NRC6. Therefore, we transiently expressed *Hero* and the 5 HCN paralogs on their own or together with *NRC6b* in leaves of the Hero introgression tomato line LA1792. As observed for the transient expression in *N. benthamiana*, none of the wildtype or autoactive HCNs induced a cell death response when expressed on their own. Cell death in tomato leaves could only be observed when the HCNs were transiently co-expressed as MHD mutant with NRC6b (Supplementary Fig. S14). Consistent with the RNA-seq expression analysis and the RT-PCRs performed, these results suggest that the NRC6 helper is not expressed in leaves under the conditions tested.

Given that *NRC6a* and *NRC6b* are exclusively expressed in roots but not in leaves, we queried our data for the other 10 NRC clade NLRs of tomato (Fig. 7C). Except for *NRC0*, *NRC1*, and *NRC3*, the other 9 NRCs displayed root-skewed expression patterns with log2-fold changes ranging from 1.33 for *NRC2* to 18 for *NRC4c* (Fig. 7C; Supplementary Table S1). Remarkably, besides *NRC6a* and *NRC6b*, *NRC4c* and *NRC5* were also exclusively expressed in roots with hardly any RNA-seq reads observed in leaves, while *NRCX* and *NRC7* are only weakly expressed in leaves compared to roots (Fig. 7; Supplementary Table S1). In addition, the strong bias towards root expression can be mapped onto the NRC phylogeny (Fig. 7C). The 5 NRCs in the large clade that comprises *NRC1*, *NRC2*, *NRC3*, and the *NRC4* clade on one hand (3 genetically linked paralogs *NRC4a*, *NRC4b* and with the exception of *NRC4c*) show expression in leaves and roots, while the genes in the phylogenetic sister clade comprising *NRC5*, *NRC6a*, *NRC6b* and *NRC7* are exclusively root expressed, or strongly skewed towards root expression (Fig. 7C; Supplementary Table S1). In summary, these findings lead us to propose that the NRC6/HCN gene cluster is exclusively functional in roots, and that root-specific expression is a feature of a number of phylogenetically related NRC helpers in the NRC receptor network.

## Discussion

**Figure 8.**
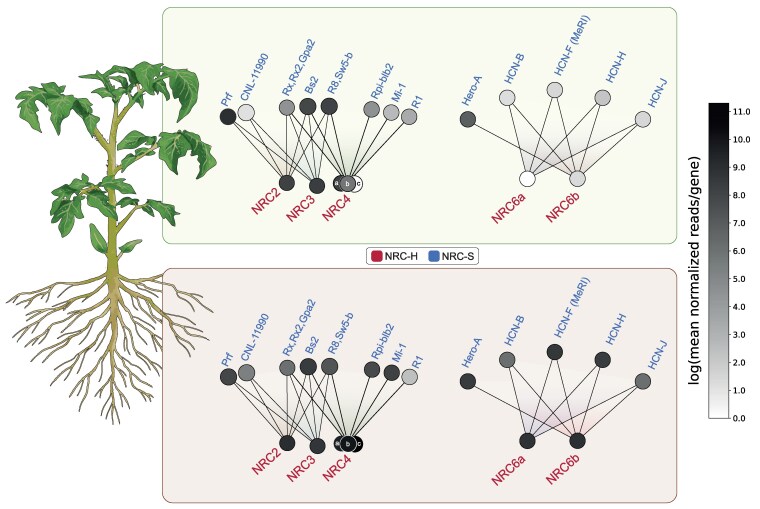
Schematic overview of organ-specific expression patterns in the NRC immune receptor network. Functional connections within the NRC network, involving known NRC sensors (NRC-S) and helpers (NRC-H), are represented based on the work of Wu et al. 2017, along with the results presented in this study. Nodes in the network are shaded to reflect their expression levels in either leaves (top) or roots (bottom), as indicated by the color scale. Expression levels are based on the mean normalized reads for each gene, derived from RNA-seq data (Supplementary Data S6) obtained from 2-week-old unchallenged tomato plants of the Hero introgression line LA1792 (Ellis and Maxon Smith 1971; Ernst et al. 2002). For NRC sensor genes from other plant species, expression data for the closest tomato homolog is displayed (Supplementary Fig. S15).

The NRC network is an intricate immune receptor network that has massively expanded in the lamiid lineage of asterid plants for over 100 million years (Wu et al. 2017; Adachi et al. 2019b; Lee et al. 2021; Goh et al. 2024). However, only a subset of the NRC proteins, which form the network's central nodes, have been functionally characterized to date. In particular, although genes in the NRC network can be genetically dispersed across the genomes of solanaceous species, such as tomato, a comprehensive analysis of genetic linkage remains outstanding. In addition, there is limited understanding of how clustered and networked NLRs are transcriptionally regulated across different plant organs. In this study, we combined genetic linkage and phylogenetic approaches to identify mixed-clade gene clusters of NLRs in tomato, revealing potential NLR sensor-helper relationships. Our analysis unveiled a mixed-clade gene cluster that is conserved across *Solanum* species and features NRC6 as a helper component and about 10 candidate sensor NLRs (HCNs). Remarkably, all gene cluster-encoded HCNs we tested in this study, including the recently identified root-knot nematode resistance gene *MeR1* and the cyst nematode resistance gene *Hero*, were dependent on *NRC6* for inducing the hypersensitive cell death. Strikingly, none of the other NRC-dependent disease resistance proteins we tested signaled through NRC6, placing this helper NLR in a distinct sub-network from NRC2, NRC3, and NRC4 (Fig. 8). Furthermore, most of the genetically linked *HCNs* and the 2 *NRC6* paralogs exhibited root-specific expression patterns across different developmental stages, suggesting that they form an organ-specific NLR network that could be implicated in resistance against root pathogens (Fig. 8). These findings offer insights into sub-functionalization within the NRC network, potentially as an adaptive response to organ-specific parasites (Fig. 8).

**Figure 9.**
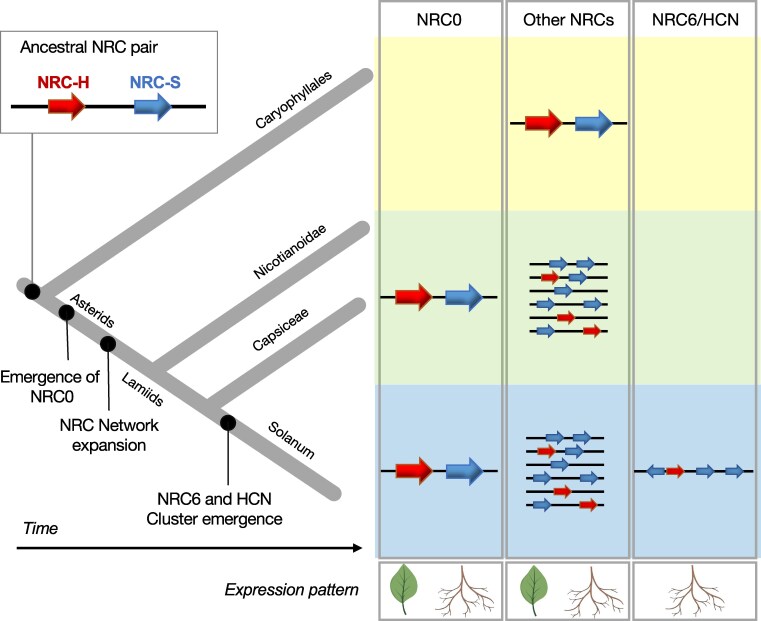
Key steps in the evolution of the NRC network. The ancestral NRC pair is thought to have emerged more than 125 million years ago (MYA) (Sakai et al. 2024). We propose that, unlike the ancient NRC0 gene cluster, the NRC6 and HCN gene cluster evolved approximately 17 to 21 MYA, prior to the diversification of the *Solanum* genus but after the lamiid expansion of the NRC network (Wu et al. 2017; Goh et al. 2024; Sakai et al. 2024). NRC6 and the HCNs formed a gene cluster through genetic duplication and diversification events during the evolution of *Solanum* species. Leaf and root symbols are used to indicate the expression pattern of the respective NLRs. NRC helpers (NRC-H), NRC sensors (NRC-S).

Our genetic clustering analysis revealed that approximately half of the tomato NLRome is encoded within gene clusters, consistent with previous observations in Arabidopsis (Meyers et al. 2003; Van de Weyer et al. 2019). While most of these gene clusters predominantly contain NLRs from the same phylogenetic clade (Meyers et al. 2003), our analysis identified mixed-clade gene clusters as well (Fig. 1, Supplementary Fig. S1). In addition to the largest gene cluster containing *NRC6* and *HCNs*, *NRC7* is also linked to an NLR gene from an NRC sensor phylogenetic clade (Figs. 1 and 2; Supplementary Data S2; Supplementary Data Set 1). Furthermore, the ancestral helper NLR NRC0, genetically clusters with NRC sensors that rely on NRC0 for the induction of cell death (Goh et al. 2024; Sakai et al. 2024; Fig. 1; Supplementary Data S2; Supplementary Data Set 1). These findings underscore that mixed-clade gene cluster configurations can be both ancient and relatively recent features of NLR evolution in asterid plants (Goh et al. 2024; Sakai et al. 2024). We propose that unlike the ancient NRC0 gene cluster, the NRC6 and HCN gene cluster has evolved about 17 to 21 MYA prior to the diversification of the *Solanum* genus, but after the lamiid expansion of the NRC network (Fig. 9).

How did the NRC6/HCN gene cluster emerge? It is plausible that NRC0, previously identified as similar to the ancestral sensor-helper NLR pair of the NRC network found in Asterids and Caryophyllales (Wu et al. 2017; Goh et al. 2024; Sakai et al. 2024), served as the progenitor of the NRC6/HCN gene cluster. This cluster could have emerged following the association of an NRC4/5-derived helper and an SD-type sensor at the same chromosomal location, possibly due to chromosomal or ectopic duplication events. Subsequently, sensor-helper specialization and tandem duplications of sensor NLRs within the gene cluster could have occurred. This scenario is supported by the close phylogenetic relation between NRC6 and NRC4/5 (Fig. 2), as well as the observation that HCNs have more recently duplicated compared with NRC6 (Seong et al. 2020). While tandem duplication events typically drive cluster expansion, other mechanisms such as unequal crossing-over, gene conversion events, or intra-cluster/gene rearrangements can also contribute to the diversification of NLR gene clusters (Noël et al. 1999; Meyers et al. 2003; Kuang et al. 2004). These mechanisms may provide insights into the wide-ranging diversity of the NRC6 and HCN gene clusters found across various *Solanum* species (Supplementary Figs. S3 and S4). Given that all HCNs tested exclusively depend on NRC6 for the induction of cell death, we hypothesize that the conserved mixed-clade cluster arrangement constitutes an evolutionary advantage by minimizing the chance of gene loss for functional sensor-helper connections during potential deleterious unequal crossing-over events (Rowan et al. 2019). It is worth noting that reconstructing the sequence of events leading to cluster formation faces significant challenges for NLR genes (Barragan and Weigel 2021). However, Seong et al. previously identified transposable elements within the NRC6/HCN cluster, suggesting that retroduplications and rearrangements within the cluster could also have shaped its composition (Seong et al. 2020).

Many NRC-dependent sensors form large gene clusters that do not contain an NRC helper (Fig. 1; Supplementary Fig. S1). An example is the root-knot nematode resistance cluster gene *Mi-1.2* (chromosome 4), which signals through the genetically unlinked helper NRC4 (Wu et al. 2017). *NRC4* forms a cluster on chromosome 4 containing 3 *NRC4* paralogs (*NRC4a/b/c*; Wu et al. 2020), which are also clustered with *NRC5*, potentially indicating that these NRCs have expanded through tandem duplication events (Fig. 1; Supplementary Fig. S1B; Wu et al. 2020). Interestingly, the *NRC4* paralogs and *NRC5* are also markedly induced in roots, relative to leaves which coincides with the implication in resistance against parasitic nematodes similar to the *NRC6* cluster (Fig. 7, Supplementary Table S1; Wu et al. 2017). One significant advantage of gene cluster formation through tandem duplications or unequal crossover events is the creation of functionally diverse NLRs (Barragan and Weigel 2021). We hypothesize that these genomic regions serve as hotspots for generating new resistance genes against pathogens, including root pathogens such as nematodes. This raises the possibility that several uncharacterized nematode, or other root pathogen, resistance genes are encoded in the NRC6/HCN gene cluster as demonstrated by the recent identification of *MeR1* (Frijters et al. 2021). It remains to be tested whether genetically clustered NLRs like *HCNs* and *Mi-1.2* can detect nematode effectors directly or by guarding a host protein targeted by plant-parasitic nematode effectors. The relatively low sequence conservation within the HCN clusters (Seong et al. 2020) may indicate a direct mode of recognition and coevolution of NLRs with pathogen effectors (Contreras et al. 2023b). Recently, a locus of hyper-variable (HYP) effector genes within *G. rostochiensis* and *G. pallida* nematode genomes has been described, potentially harboring hundreds of allelic effector variants (Sonawala et al. 2024). The existence of such gene clusters may explain why *Solanum* plant genomes evolved to encode extended, variable clusters of root-expressed NLR genes as a counter-measure.

Rather than being uniformly expressed, NLR genes can show organ-, tissue-, or cell-specific expression patterns, which may reflect adaptations to distinct pathogen pressures (Munch et al. 2018; Tang et al. 2023). However, reports on specific expression patterns and functionality remain scarce. Interestingly, all HCNs, including the nematode resistance genes *Hero* and *MeR1*, as well as several helper NLRs of the NRC clade exhibited a marked root-skewed baseline expression pattern in tomato (Figs. 7 and 8, Supplementary Table S1). Although some of the *HCNs*, including *Hero*, show low expression in leaves (Sobczak et al. 2005), the *NRC6* helpers are exclusively expressed in roots across different developmental stages (Fig. 7; Supplementary Figs. S12 and S13). Our results demonstrate that Hero, MeR1, and the tested HCNs specifically depend on NRC6 for the induction of cell death (Figs. 3 to 5; Supplementary Fig. S14). This outlines that the functionality of NRC sensor clade resistance genes is dependent on the expression profile of the corresponding NRC helper(s).

Apart from the root-specific *NRC6*, the phylogenetically related *NRC5* and *NRC7* are strongly expressed in roots (Fig. 7). Moreover, *NRC4* paralogs show robust expression in roots (Figs. 7 and 8; Supplementary Table S1). Several NLRs in the NRC sensor clade also display a root-specific expression pattern, revealing them as potential resistance genes that might signal through root-specific or root-expressed helpers, and might act against root pathogens (Fig. 7A, Supplementary Fig. S15). This observed expression pattern in tomato further aligns with the root-skewed NLR expression previously noted in multiple plant species (Gao et al. 2013; Millett et al. 2015; Deng et al. 2017; Munch et al. 2018; Barbey et al. 2019; Adachi et al. 2023). It suggests that the plant immune system may have evolved specialized defenses tailored to organ-specific pathogens (Bhardwaj et al. 2011; Wang et al. 2011; Munch et al. 2018; Adachi et al. 2019b; Tang et al. 2023).

Could the organ-specific expression of NLR genes be driven by an evolutionary trend to evade autoimmunity? Elevated expression of NLR genes can potentially harm plant fitness by increasing the risk of unintended activation (Bomblies et al. 2007; Yi and Richards 2009; Karasov et al. 2017). Organ-specific transcriptional regulation is a potential mechanism to mitigate the risk of inadvertent NLR activation, thereby reducing the fitness penalties that may occur when new sensor NLR alleles emerge to detect pathogen effectors. Indeed, the presence of an NLR gene can have a significant negative effect on plant fitness in the absence of infection (Tian et al. 2003; Karasov et al. 2014). In addition, elevated expression or misregulation of NLRs has been shown induce autoimmune phenotypes (Palma et al. 2010). Uncoupling expression between organs or tissues might, for example, offer a means to avoid NLR autoactivity or fitness costs in leaves while maintaining baseline expression levels of sensors in roots or other organs targeted by specific pathogens. HCN-B and MeR1 exhibit autoimmunity when co-expressed as wildtype variants with NRC6 in the leaves of *N. benthamiana* (Figs. 3 and 6). However, no autoimmunity was observed when HCN-B was transiently expressed in tomato leaves (Supplementary Fig. S14), suggesting that there might also be additional mechanisms in host plants that suppress the autoactivity of sensor-helper pairs.

How is organ specificity achieved? One way to establish organ-specific expression patterns is through the control of gene expression, using specific transcriptional elements (Dutt et al. 2014). Investigating common promoter motifs, transcription factor binding sites, or the epigenetic regulation of these gene clusters may provide additional insights into the generation of these organ-specific gene expression patterns (Deng et al. 2017; Bartels et al. 2018; Zhou et al. 2022). It is worth noting that NLRs, including those in the tomato NLRome, are also regulated at the transcript level by small RNAs (Zhai et al. 2011). However, our early investigations have not revealed any particularly obvious patterns in the NRC6/HCN cluster genes that would provide cues about root-specific expression. Further research is needed to determine whether the generation of organ- or tissue-specific small RNAs contributes to the observed differences in transcript levels.

The organ-specific facet of the NRC network we documented here has implications for the identification and deployment of disease resistance in agriculture. Considering that plant parasites often infect specific organs and tissues, the expression patterns of NLR genes can accelerate the cloning of additional disease resistance genes by reducing the number of potential candidate genes. Additionally, the development of bioengineered NLRs for resistance against specific pathogens may benefit from organ-specific expression patterns to ensure effective immunity while mitigating the risk of autoimmune responses (Adachi et al. 2019b; Kourelis et al. 2023; Contreras et al. 2023a).

## Materials and methods

### Gene cluster analysis

Gene cluster analysis was performed as described previously (Sakai et al. 2024). Briefly, the NLR gene annotations were extracted from the ITAG3.2 and RefSeq SL3.0 gff3 files using NLRtracker (Kourelis et al. 2021) and the distance between each NLR gene was determined by a custom script (https://github.com/slt666666/NRC0). NLRs were considered as clustered when the genetic distance was less than or equal to 50 kb. The phylogenetic relationship was based on the NB-ARC domain of extracted sequences and the genetic distance matrix shown in Fig. 1 and was generated by using a previously developed “gene cluster-matrix” library (https://github.com/slt666666/gene-cluster-matrix). All sequences, alignments, phylogenetic tree files, and the interactive matrix file are available as Supplementary Data Set 1.

### Database search for NRC6 and HCN homologs

The full-length NLR sequences retrieved from the NLRtracker (Kourelis et al. 2021) output of 124 Solanaceae genomes (https://doi.org/10.5281/zenodo.10354350 (Sugihara et al. 2023)) were filtered for sequences with “CNL” and “BCNL” domain architectures. The NB-ARC domain was extracted from the identified 21.833 sequences. Domains shorter than 300 or longer than 400 amino acids were removed as a length distribution parameter applied across all NB-ARC domains. The NB-ARC domains from the RefPlantNLR (Kourelis et al. 2021) dataset, the NRCX dataset (Adachi et al. 2023), HCNs, and tomato (*Solanum lycopersicum*) NRCs were incorporated, resulting in a refined dataset containing 20.292 sequences. The NB-ARC domains were aligned using MAFFT v7.505 (Katoh et al. 2002; Katoh and Standley 2013) with the [–anysymbol] option, and a phylogenetic tree was constructed using FastTree v2.1.10 (Price et al. 2010) with the [-lg] option. A custom R script was employed for sub setting of well-supported major branches to generate specific sub-trees for NRC helper, NRC-SD sensor, and HCN clades. The resulting phylogenetic trees were visualized using iTOL (Letunic and Bork 2021) and manually annotated. All scripts utilized in this analysis are deposited under https://github.com/amiralito/Hero (Toghani et al. 2025). All sequences, alignments and phylogenetic tree files are available as Supplementary Data Set 2 or Supplementary Data Set 3.

### Determination of NRC6 and HCN gene clusters from genome-scale assemblies of Solanum plants

BLAST (Altschul et al. 1990) searches for Hero and NRC6 homologs were performed in Geneious Prime (https://www.geneious.com), against local databases of genome sequences and annotations of wild tomato (*S. pennelli*, SPENNV200, Bolger et al. 2014), potato (*S. tuberosum*, DM 1-3 516 R44, Potato Genome Sequencing Consortium et al. 2011), and American black nightshade (*S. Americanum*, SP2271, Lin et al. 2023). Genes in the genomic loci of the received top hits were extracted, translated, and annotated to verify the presence of an NB-ARC domain (Pfam ID: PF00931). The detected NLR gene clusters were drawn to scale.

### Plant growth condition

For cell death assays, wildtype and *nrc2/3/4* mutant (Wu et al. 2020) *Nicotiana. benthamiana* plants and the Hero tomato line (*S. lycopersicum*, LA1792) were sown on soil (Levington F2 Starter Compost) and grown in a glasshouse. Wildtype *N. benthamiana* plants used for protein extraction, and the Hero tomato line (*S. lycopersicum*, LA1792) used for RNA-extraction and RNA-seq were sown on soil (Levington F2 Starter Compost) and grown in a growth chamber at 22 to 25 °C, 45% to 65% humidity, and a 16 h /8 h light/dark cycle.

### Plasmid construction

The Golden Gate Modular Cloning (MoClo; Weber et al. 2011) and MoClo plant parts kits (Engler et al. 2014) were used for cloning. Wildtype and MHD mutant variants of tomato NRC helpers and HCNs were synthesized as *N. benthamiana* codon-optimized L0 modules in pICH41155 through GENEWIZ/AZENTA (https://www.azenta.com/). All plasmids generated in this study were cloned into the binary vector pJK001c (Paulus et al. 2020). Cloning design and sequence analysis were performed in Geneious Prime (https://www.geneious.com). All Plasmids used and constructed in this study are described in Supplementary Data S7.

### Generation of the *Rhizobium rhizogenes* A4-derived strain AS107

The *Rhizobium rhizogenes* A4-derived strain AS107 was generated using the INTEGRATE system in the AS101 background (Vo et al. 2021; Aliu et al. 2022; Lopez-Agudelo et al. 2025). In brief, the spacer sequence targeting the putative kanamycin resistance genes in the chromosomal background of A4 was inserted into pEA186 (Addgene #187874). This plasmid was then electroporated into AS101 and plated on 523 medium (0.8% casein hydrolysate (N-Z-Case), 0.4% yeast extract, 0.2% K_2_HPO_4_, and 0.03% MgSO_4_·7H_2_O, adjusted to pH 7) supplemented with glucose (0.25%) and spectinomycin (200 mg/L). The resulting colonies were subcultured in fresh 523 medium supplemented with glucose (0.25%) and spectinomycin (200 mg/L) until pure colonies of edited bacteria were identified. These colonies were subsequently plated on 523 medium supplemented with 5% sucrose to remove the plasmid pEA186. Successful deletion of the kanamycin resistance gene was confirmed via Sanger sequencing. Rifampicin resistance was then introduced by growing the bacteria in liquid 523 medium supplemented with 1% sucrose and rifampicin (0.5 mg/L). The bacteria were subcultured into the same medium with rifampicin concentrations incrementally increased to 1, 2, 5, 10 mg/L, and finally 50 mg/L.

### Transient gene expression and cell death assays

Transient gene expression in *N. benthamiana* was performed by agroinfiltration. *Agrobacterium tumefaciens* strain GV3101 pMP90 transformed with respective binary expression constructs were inoculated from glycerol stocks and grown O/N at 28 °C in LB supplemented with appropriate antibiotics. For transient gene expression in tomato, *R. rhizogenes* strain AS107 transformed with respective binary expression constructs were inoculated from glycerol stocks and grown O/N at 28 °C in 523-medium supplemented with appropriate antibiotics. Cells were harvested by centrifugation at 2,000 × g for 10 min at RT and resuspended in infiltration buffer (10 mm MgCl_2_, 10 mm MES-KOH pH 5.6, 200 μM acetosyringone). Cells were left to incubate in the dark for 2 h at room temperature prior to infiltration into 5- to 6-week-old *N. benthamiana* leaves or tomato leaflets at ODs indicated in Supplementary Data S7, respectively. Cell death phenotypes for *N. benthamiana* were scored with a range from 0 (no visible necrosis) to 7 (fully confluent necrosis) according to (Adachi et al. 2019a). Quantification and statistical analysis was performed by using the besthr R library (MacLean 2019) and plotted using a script described in Bentham et al. 2023. Scoring for all experiments can be found in Supplementary Data Set 4.

### Protein extraction and SDS-PAGE assay

*N. benthamiana* leaf discs (8 mm diameter) were taken 2 days post-infiltration (dpi) with Agrobacteria and were homogenized in extraction buffer [10% glycerol, 25 mm Tris-HCl, pH 7.5, 1 mm EDTA, 150 mm NaCl, 1% (w/v) PVPP, 10 mm DTT, 1 × protease inhibitor cocktail (SIGMA), 0.2% IGEPAL CA-630 (SIGMA)]. After centrifugation at 12,000 × g for 10 min at 4 °C, the obtained supernatant was mixed with 2 × SDS loading buffer [final concentration: 50 mm Tris-HCl (pH 6.8), 100 mm DTT, 2% SDS, 0.01% bromophenol blue, 10% glycerol] and denatured at 72 °C for 10 min. Total protein extracts were separated by SDS-PAGE gels (Bio-Rad) and transferred onto polyvinylidene difluoride (PVDF) membranes using a Trans-Blot turbo transfer system (Bio-Rad). Membranes were blocked for 60 min in 5% milk powder dissolved in Tris-buffered Saline [50 mm Tris-HCL (pH7.5), 150 mm NaCl]. Mouse monoclonal anti-GFP antibody conjugated to HRP (B-2, Santa Cruz Biotech; 1:5,000 dilution) or mouse monoclonal anti-mCherry TrueMAB antibody conjugated to HRP (OTI10G6, Thermo Fisher Scientific; 1:2,500 dilution) were used to probe the membranes. Equal loading was monitored by staining the PVDF membranes with Ponceau S (SIGMA).

### RNA-extraction, RNA-seq analysis and HCN reading frame adjustment

Total RNA from leaf and root tissues for RNA-seq of 2-week-old *S. lycopersicum*, LA1792 was extracted from independent biological triplicates using the RNeasy Mini Kit (Qiagen). Each sample was sent for library preparation and Illumina NovaSeq 6000 sequencing (40 m paired-end reads per sample, Novogene). High-quality reads were mapped to a pseudogenome generated by replacing the NRC6 and HCN gene cluster of the Heinz reference assembly (SL3.0, ITAG3.2 annotation) with the cluster sequence obtained by (Ernst et al. 2002), using HISAT2 (Kim et al. 2019). Read alignment to the NRC6 and HCN gene cluster was used to correct reading frames and sequences of NRC and HCN gene cluster-encoded genes as an iterative approach. The CDS sequences of HCN-B (OR865987), HCN-C (OR865986), HCN-F (OR865983), HCN-H (OR865981), HCN-J (OR865982), NRC6a (OR865985), and NRC6b (OR865984) were deposited to the National Center for Biotechnology Information (NCBI) GenBank. Read counting was performed using STRINGTIE2 (Pertea et al. 2015; Kovaka et al. 2019) and differential expression of genes was determined by DeSeq2 (Love et al. 2014), comparing leaf against root tissues. Raw reads used in this study were deposited in the NCBI Sequence Read Archive (SRA) with BioSample accession SAMN38499990 and BioProject accession PRJNA1046475. The pseudogenome, pseudoannotation, original NRC6/HCN cluster sequences generated by Ernst et al. 2002, as well as the corrected cluster sequences and annotations are available under https://zenodo.org/records/10376142 (Lüdke et al. 2023). Scripts used for RNA-seq analysis are deposited under https://github.com/amiralito/Hero (Toghani et al. 2025).

### RT-PCR

Leaf and root tissues for RT-PCRs of 3-, 4-, 5-, or 6-week-old *S. lycopersicum*, LA1792 were collected from 3 independent plants and pooled before using the RNeasy Mini Kit (Qiagen) to extract total RNA. The SuperScript IV VILO Master Mix with ezDNAase (Thermo Fisher Scientific) was used according to manufacturer's instructions to generate cDNA. RT-PCRs were performed with DreamTaq DNA Polymerase (Thermo Fisher Scientific) and intron spanning, cDNA specific primers listed in Supplementary Data S8, according to manufacturer's instructions.

### Accession numbers

Sequence data from this article can be found in the GenBank libraries under accession numbers: Hero (AJ457051); HCN-B (OR865987); HCN-C (OR865986); HCN-F (OR865983); HCN-H (OR865981); HCN-J (OR865982); NRC6a (OR865985); NRC6b (OR865984).

## Supplementary Material

koaf145_Supplementary_Data

## Data Availability

All data is available in the manuscript and the supplementary data or under https://github.com/amiralito/Hero (Toghani et al. 2025), https://zenodo.org/records/10376142 (Lüdke et al. 2023) and https://doi.org/10.5281/zenodo.10354350 (Sugihara et al. 2023).

## Supplementary data

The following materials are available in the online version of this article.

**Supplementary Figure S1.** The tomato genome encodes homo and mixed-clade NLR gene clusters with different compositions.

**Supplementary Figure S2.** NRC6 is a canonical NRC helper with an extension before the MADA motif.

**Supplementary Figure S3.** The NRC6/HCN gene cluster emerged in *Solanum* plants.

**Supplementary Figure S4.** Comparison of the NRC6/HCN gene cluster in *Solanum* species.

**Supplementary Figure S5.** Quantification and statistical analysis of cell death upon co-expression of HCNs with NRC6a or NRC6b.

**Supplementary Figure S6.** Accumulation of wildtype and autoactive HCNs in planta.

**Supplementary Figure S7.** Quantification and statistical analysis of cell death upon co-expression of NRC2/3/4-dependent sensors.

**Supplementary Figure S8.** Quantification and statistical analysis of cell death upon co-expression of autoactive HCNs and tomato NRCs.

**Supplementary Figure S9.** Tomato NRC helpers accumulate in planta.

**Supplementary Figure S10.** Quantification and statistical analysis of cell death upon co-expression of wildtype or autoactive MeR1 and NRC6 or other tomato NRC helpers.

**Supplementary Figure S11.** The RNA-seq data of leaf and root tissues are consistent across the 3 biological replicates.

**Supplementary Figure S12.** Expression profile of NRC6/HCN cluster genes in the Heinz reference tomato line.

**Supplementary Figure S13.** HCNs and NRC6 helper genes are nearly exclusively expressed in tomato roots across developmental stages.

**Supplementary Figure S14.** HCNs require NRC6b to trigger hypersensitive cell death when transiently expressed in tomato leaves.

**Supplementary Figure S15.** Resistance gene homologs in tomato show varying degrees of expression in roots and leaves.

**Supplementary Table S1.** Expression and log2-fold change of HCN and NRC genes in tomato roots and leaves.

**Supplementary Data S1.** Tomato NLR gene IDs and NLR phylogenetic subclades.

**Supplementary Data S2.** Tomato NLR gene distance matrix.

**Supplementary Data S3.** Overview and summary of NLR gene clusters in tomato.

**Supplementary Data S4.** Genomes used for NRC6 and HCN homolog detection for Fig. 2.

**Supplementary Data S5.** Log2-fold changes between leaf and root tissues detected by RNA-seq.

**Supplementary Data S6.** Raw and normalized RNA-seq counts of leaf and root tissues.

**Supplementary Data S7.** Plasmids used and constructed in this study.

**Supplementary Data S8.** Primers used in this study.

**Supplementary Data Set 1.** Distance file, alignment and phylogenetic tree files used for Fig. 1.

**Supplementary Data Set 2.** Sequence, alignment, and phylogenetic tree files used for Fig. 2.

**Supplementary Data Set 3.** Sequence, alignment, and phylogenetic tree files used for Fig. 6.

**Supplementary Data Set 4.** HR scoring data used for Figs. 3 to 6.

## Dive Curated Terms

The following phenotypic, genotypic, and functional terms are of significance to the work described in this paper:

NCBI Gramene: https://www.ncbi.nlm.nih.gov
NCBI Araport: https://www.ncbi.nlm.nih.gov

ZAR1 Gramene: AT3G50950
ZAR1 Araport: AT3G50950
