# Supplementary material for: A root-specific NLR network mediates immune signaling of resistance genes against plant parasitic nematodes
Source: Plant Cell. 2025 Jun 24;37(7):koaf145. doi: 10.1093/plcell/koaf145 (PMC12236159; doi:10.1093/plcell/koaf145)
Supplement: koaf145_Supplementary_Data [file koaf145_supplementary_data.zip › TPC-2025-0377_Supplementary_Figures and Table.pdf]

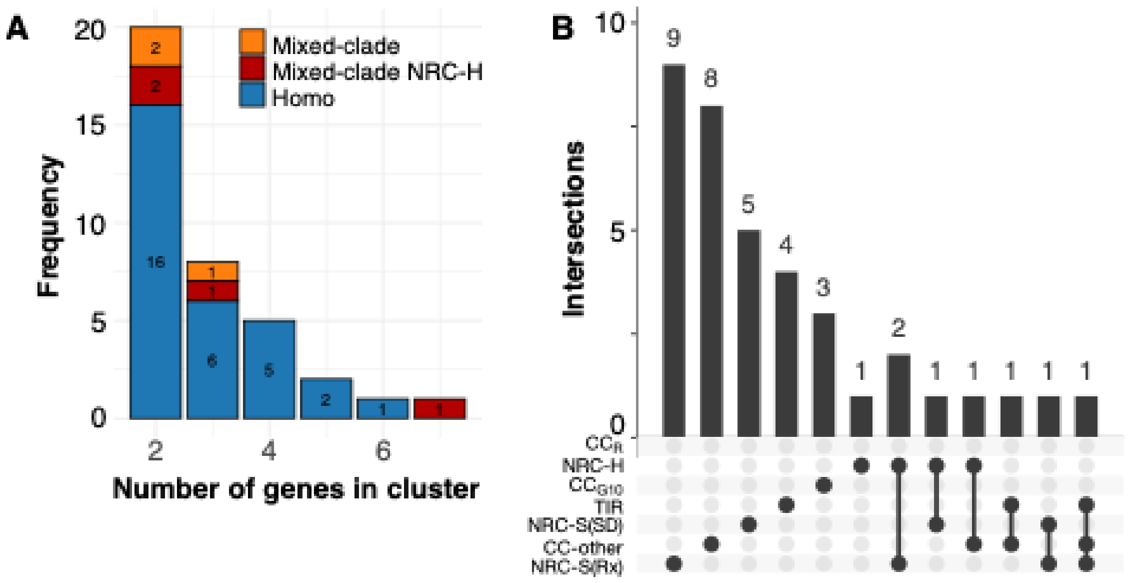

Supplementary Figure S1. The tomato genome encodes homo and mixed-clade NLR gene clusters with different compositions. (**A**) Frequency of NLR gene cluster sizes found in the tomato genome. The number of homo (blue), mixed-clade gene clusters (orange), and mixed-clade gene clusters containing NRC helpers (red) are indicated. (**B**) Upset plot showing the frequency for cluster compositions for all detected tomato NLR gene clusters. The frequency of homo clusters for respective NLR clades are indicated by a single dot, frequency of mixed-clade gene clusters and the respective composition is indicated by dots joined together by a line. Coiled-Coil-type (CC), RESISTANCE TO POWDERY MILDEW 8 (RPW8)-type (CC_R), G10-type CC (CC_{G10}), other CC-type (CC-other), Toll/interleukin-1 receptor-type (TIR), Rx-type NRC sensors ($NRC\text{-}S_{(Rx)}$), Solanaceous domain (SD)-type NRC sensors ($NRC\text{-}_{(SD)}$), NRC helpers (NRC-H).

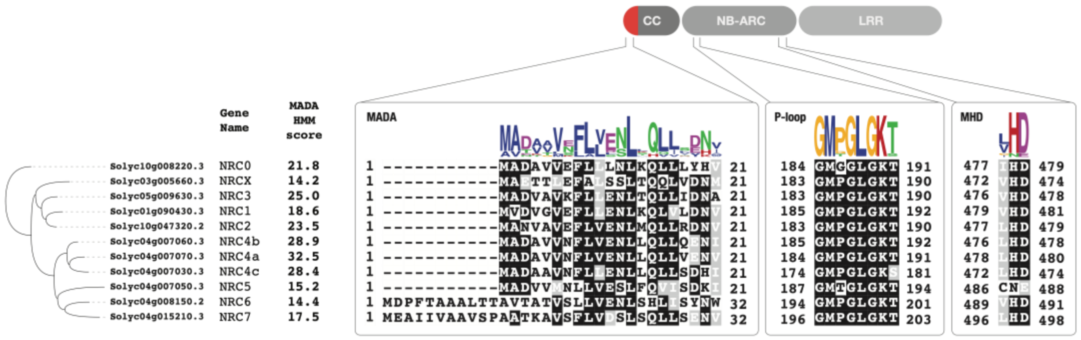

Supplementary Figure S2. NRC6 is a canonical NRC helper with an extension before the MADA motif. Schematic representation of the NRC helper domain architecture (top), depicting Coild-coil (CC), Nucleotide-binding adaptor shared by APAF-1, certain R gene products, and CED-4 (NB-ARC), and Leucine-rich repeat (LRR) domains. The position of the α1 helix within the CC domain, containing the MADA motif, is indicated in red. The MADA motif score, based on a Hidden-Markov model (Adachi et al. 2019a), is shown for each tomato NRC helper. A MEME motif was generated for the MADA motif, the P-loop motif, and the MHD motif based on the amino acid sequence alignment of all tomato NRC helpers. The respective amino acid positions of the motifs within each NRC helper are indicated. Black shading indicates conserved residues; grey shading indicates similar residues. In the logo plot, blue, green, magenta, red, pink, and orange, are used to indicate hydrophobic, polar, acidic, basic, weakly basic, or small amino acids respectively.

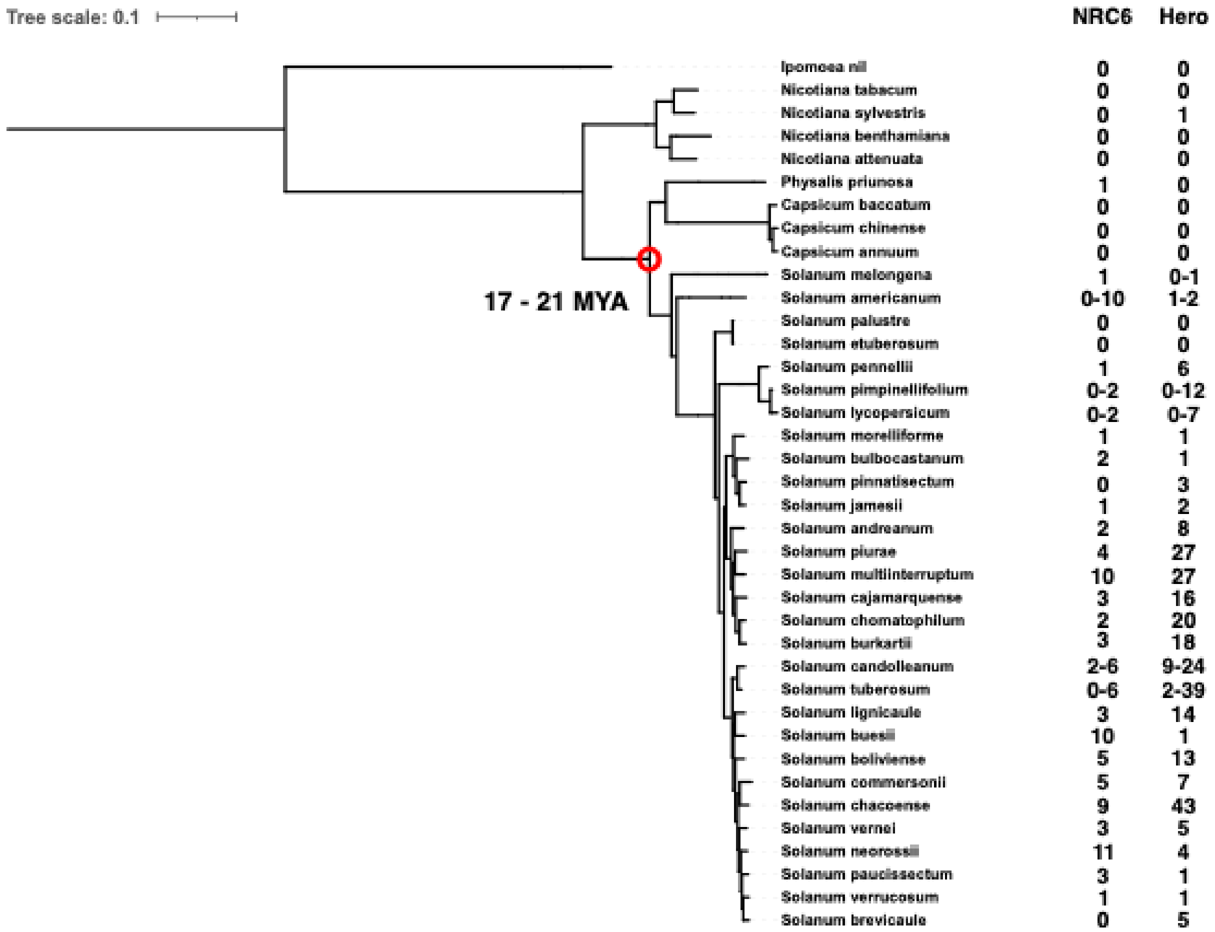

Supplementary Figure S3. The NRC6/HCN gene cluster emerged in *Solanum* plants. A phylogenetic tree illustrating the species relationships between Caryophyllales and asterid plant species is presented. NRC6, along with Hero-homologs, is exclusively encoded within Solanum plants (Supplementary Data 4). The range of numbers for NRC6 and Hero-homologs in each species is based on phylogeny, using the data displayed in Figure 2 and Supplementary Dataset 2. The species phylogeny was derived from (Wu et al., 2023), the time of divergence is based on (Särkinen et al. 2013) provided in million years (MYA). The scale bar represents evolutionary distance, measured as substitutions per site.

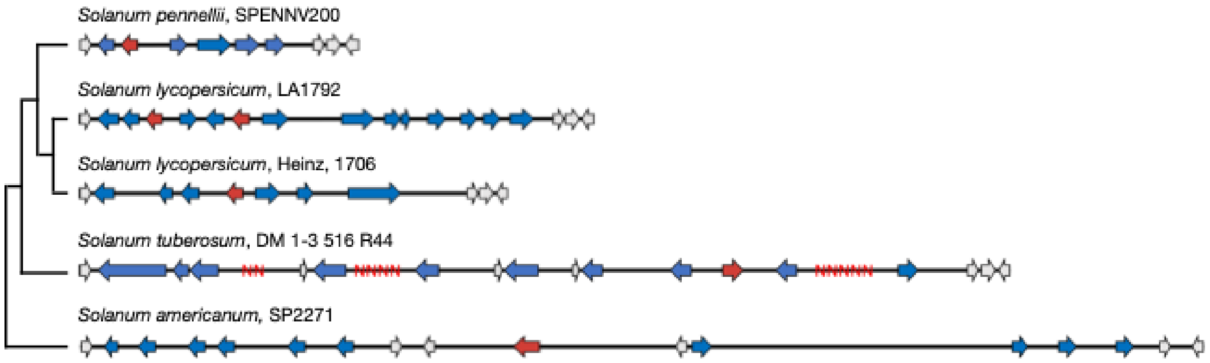

Supplementary Figure S4. Comparison of the NRC6/HCN gene cluster in *Solanum* species. Schematic representation of the NRC6 and HCN gene cluster extracted from chromosome-scale genome sequence assemblies of wild tomato (*S. pennelli*), potato (*S. tuberosum*), and American black nightshade (*S. americanum*), together with the gene clusters of *S. lycopersicum* Heinz 1706 and Hero LA1792, which contains an introgressed cluster from *S. pimpinellifolium* LA121 (Ellis and Maxon Smith 1971; Ernst et al. 2002). The NRC6 helper is colored in red, HCNs are colored in blue, and non-NLRs are colored in gray. Gaps in the sequence assembly are depicted as red Ns.

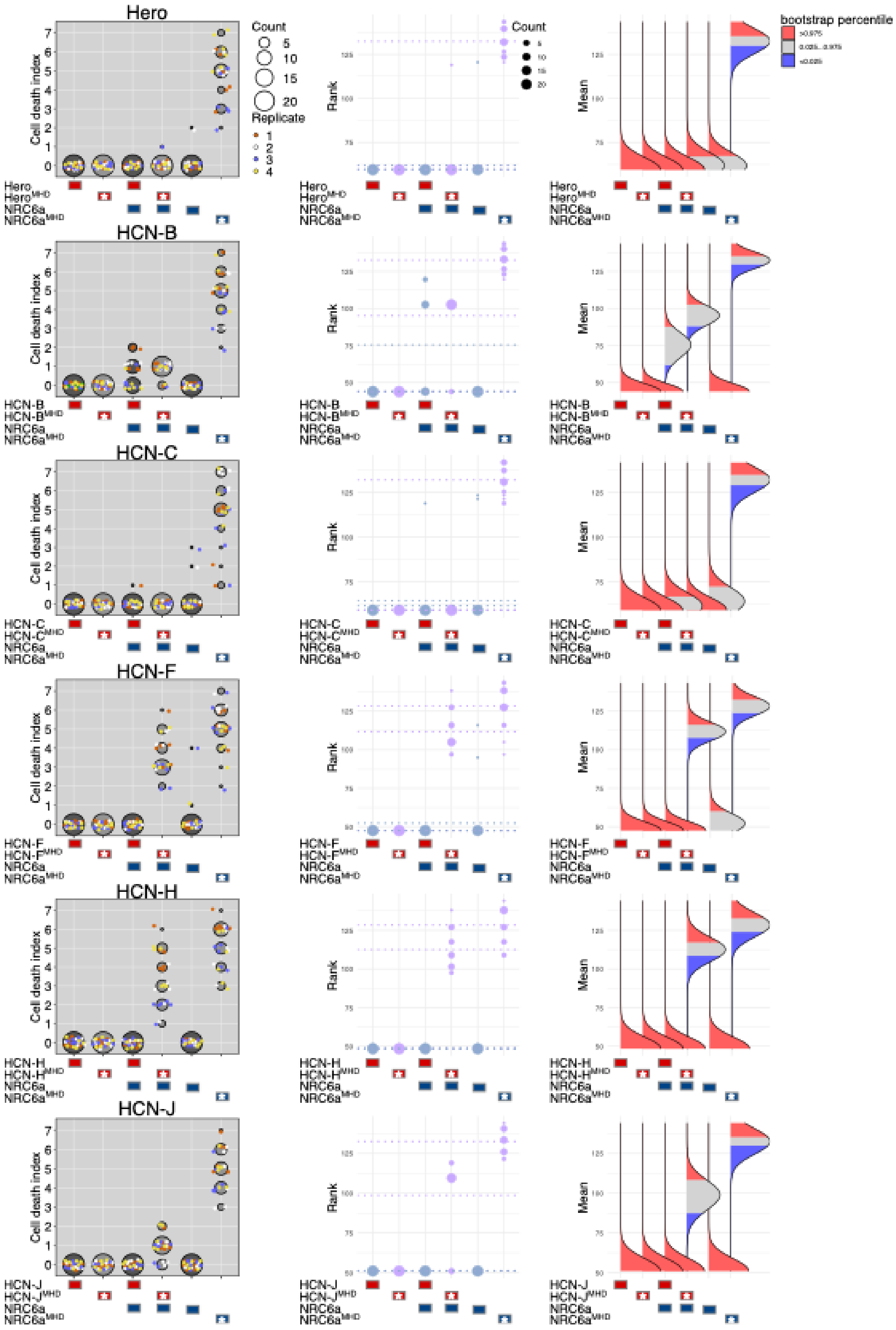

Hero
HCN-B
HCN-C
HCN-F
HCN-H
HCN-J
Cell death index
Count
5
10
15
20
Replicate
1
2
3
4
Rank
Mean
bootstrap percentile
Hero
Hero^MHD
HCN-B
HCN-B^MHD
HCN-C
HCN-C^MHD
HCN-F
HCN-F^MHD
HCN-H
HCN-H^MHD
HCN-J
HCN-J^MHD
NRC6a
NRC6a^MHD

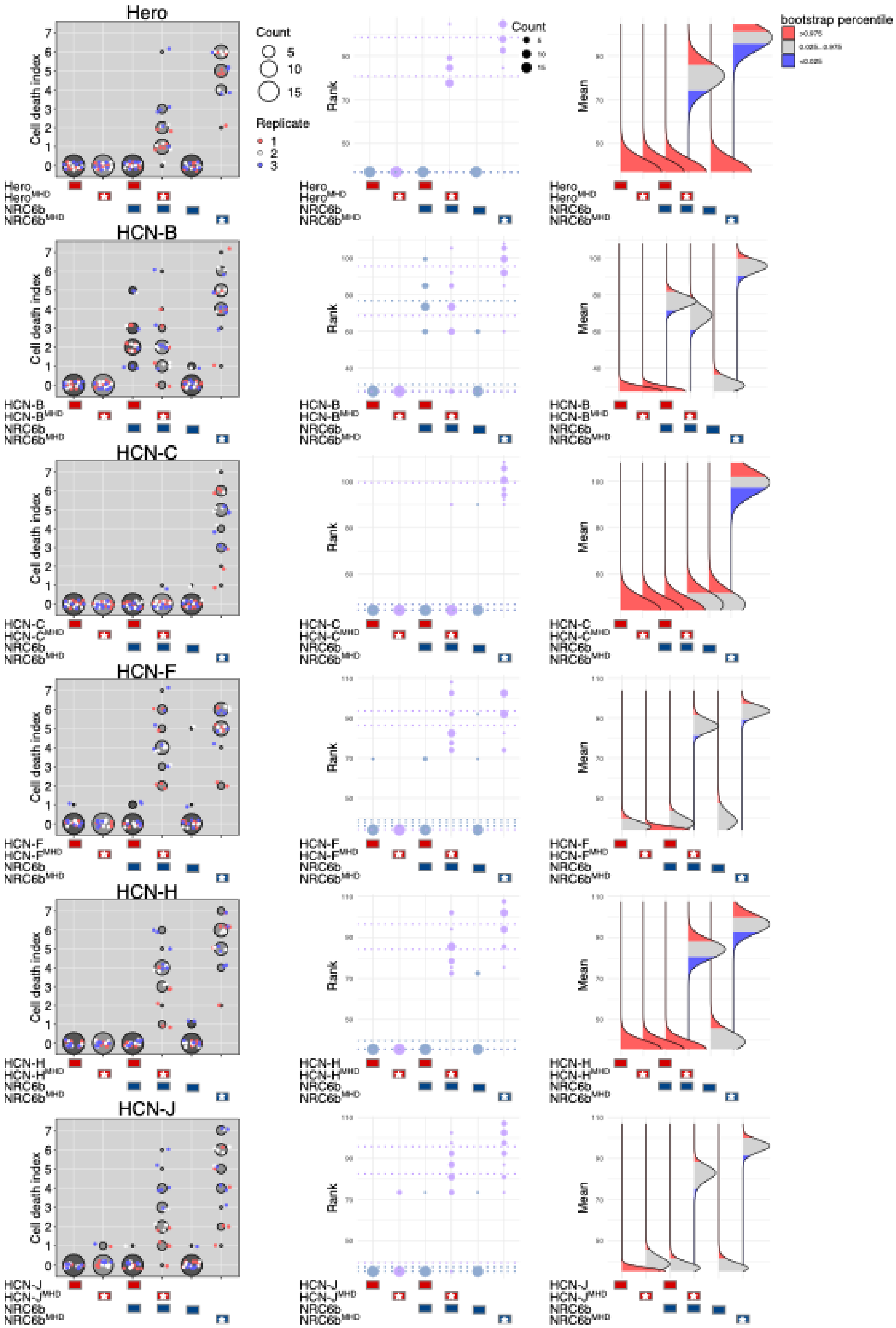
Hero
HCN-B
HCN-C
HCN-F
HCN-H
HCN-J
Cell death index
Count
5
10
15
Replicate
1
2
3
Rank
Mean
bootstrap percentile
Hero
Hero^MHD
NRC6b
NRC6b^MHD
HCN-B
HCN-B^MHD
HCN-C
HCN-C^MHD
HCN-F
HCN-F^MHD
HCN-H
HCN-H^MHD
HCN-J
HCN-J^MHD

Supplementary Figure S5. Quantification and statistical analysis of cell death upon co-expression of HCNs with NRC6a or NRC6b. (left) Cell death data represented as dots, colored for each biological replicate. The central circle for each cell death category proportionally represents the total number of data points. An asterisk indicates expression of an autoactive NLR MHD mutant (denoted as MHD). Each biological replicate consists of two leaves from three different plants each. **(middle)** Statistical analysis using the besthr R library (MacLean, 2019). The ranked data is shown as dots with their corresponding mean as dashed line. The size of each dot proportionally represents the total number of data points. A bootstrap resampling test, using a lower significance cutoff of 0.025 and an upper cutoff of 0.975, was performed. Mean ranks of test samples falling outside of these cutoffs in the control samples bootstrap population (respective HCN alone) were considered significant. **(right)** The distribution of 1,000 bootstrap sample rank means, blue areas under the curve illustrate the 0.025, and red areas the 0.975 percentiles of the distribution.

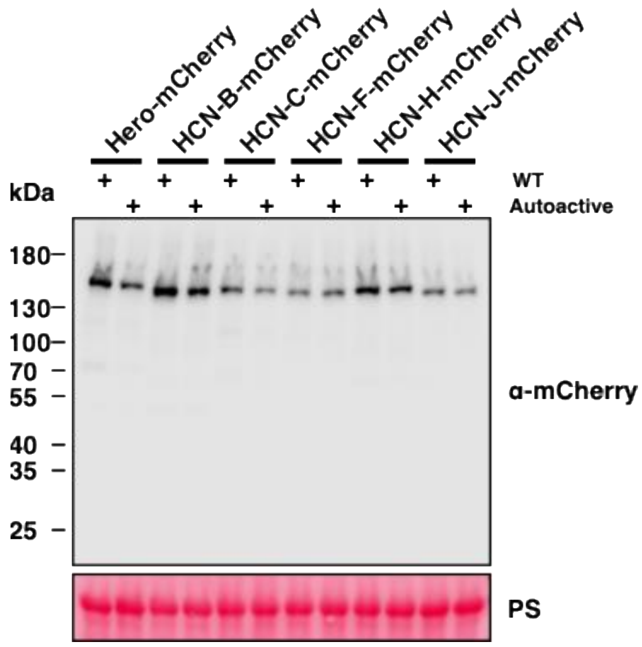

Supplementary Figure S6. Accumulation of wild-type and autoactive HCNs in planta. Immunoblot analysis depicting the accumulation of HCN-mCherry in wildtype *N. benthamiana* plants at 2 days post-infiltration (dpi) with Agrobacteria transformed with the respective expression constructs, a p19 silencing construct was co-expressed for each infiltration. Ponceau S stain (PS).

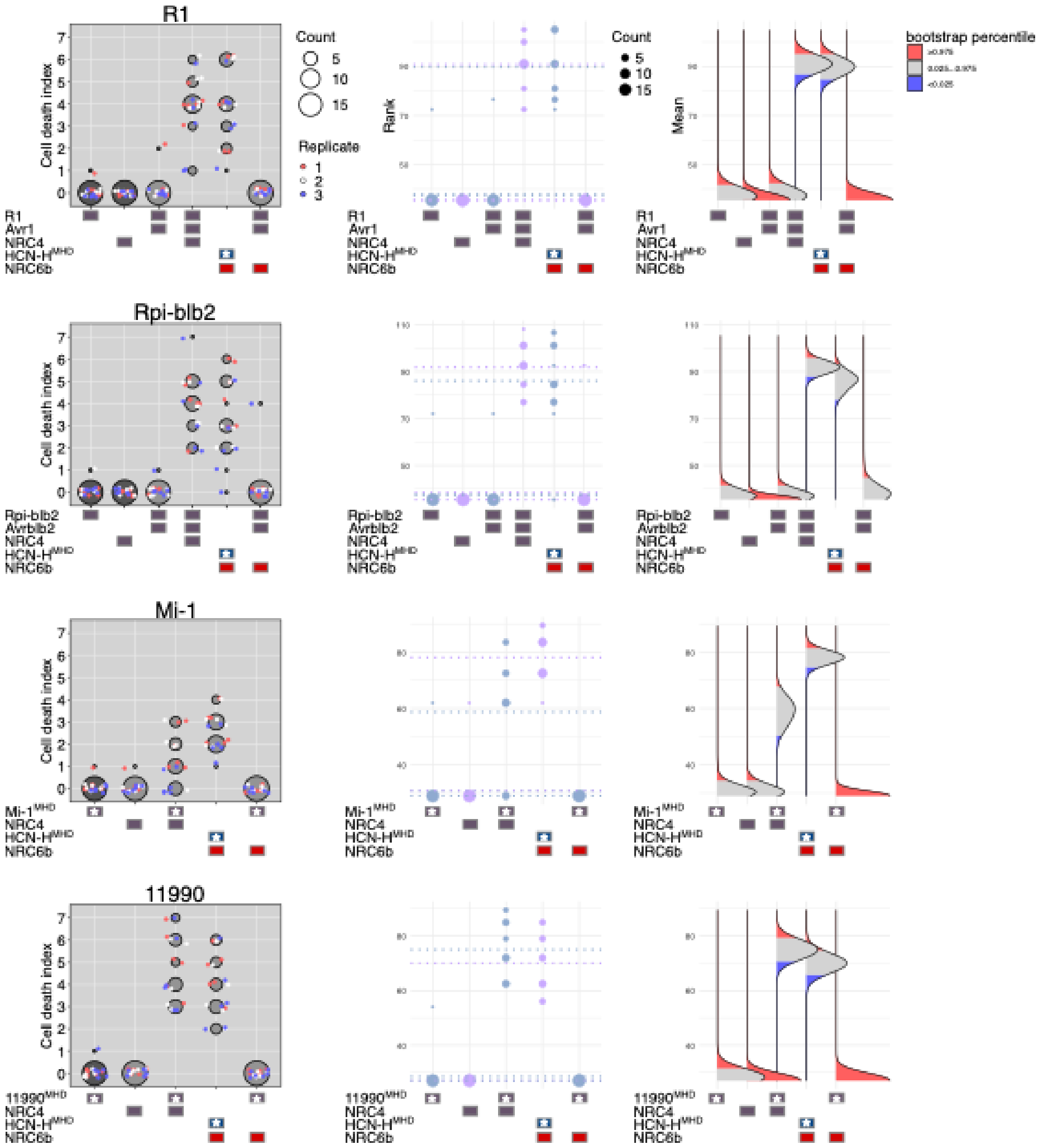

R1
Rpi-blb2
Mi-1
11990
Cell death index
Rank
Mean
Count
Replicate
bootstrap percentile
R1
Avr1
NRC4
HCN-H^MHD
NRC6b
Rpi-blb2
Avrblb2
Mi-1^MHD
11990^MHD

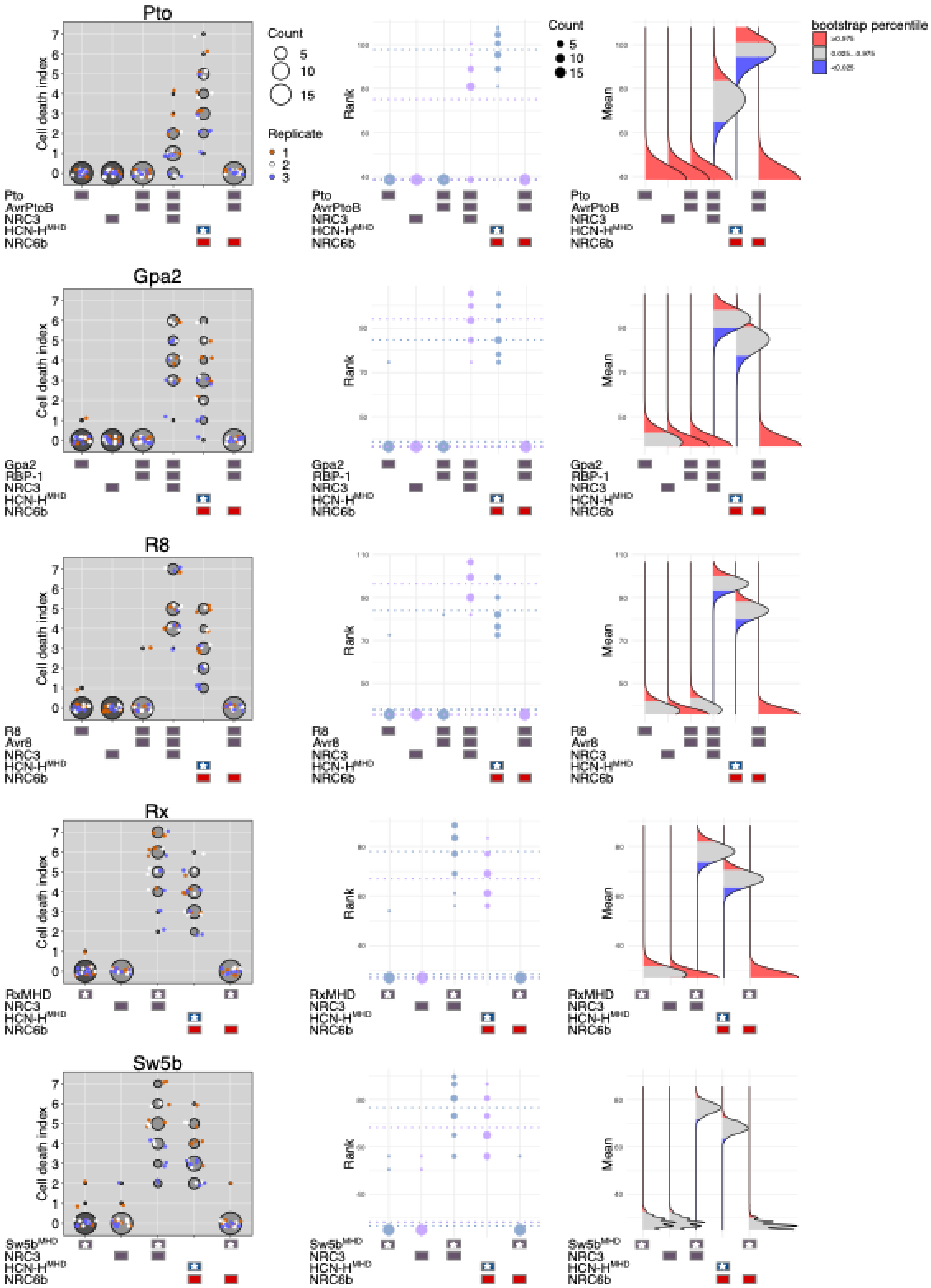
Pto
Count
5
10
15
Replicate
1
2
3
Cell death index
Rank
Mean
bootstrap percentile
Pto
AvrPtoB
NRC3
HCN-H^MHD
NRC6b
Gpa2
RBP-1
NRC3
HCN-H^MHD
NRC6b
R8
Avr8
NRC3
HCN-H^MHD
NRC6b
Rx
RxMHD
NRC3
HCN-H^MHD
NRC6b
Sw5b
Sw5b^MHD
NRC3
HCN-H^MHD
NRC6b

Supplementary Figure S7. Quantification and statistical analysis of cell death upon co-expression of NRC2/3/4-dependent sensors. (left) Cell death data represented as dots, colored for each biological replicate. The central circle for each cell death category proportionally represents the total number of data points. An asterisk indicates expression of an autoactive NLR MHD mutant (denoted as MHD). Each biological replicate consists of two leaves from three different plants each. **(middle)** Statistical analysis using the besthr R library (MacLean, 2019). The ranked data is shown as dots with their corresponding mean as dashed line. The size of each dot proportionally represents the total number of data points. A bootstrap resampling test, using a lower significance cutoff of 0.025 and an upper cutoff of 0.975, was performed. Mean ranks of test samples falling outside of these cutoffs in the control samples bootstrap population (sensor alone) were considered significant. **(right)** The distribution of 1,000 bootstrap sample rank means, blue areas under the curve illustrate the 0.025, and red areas the 0.975 percentiles of the distribution.

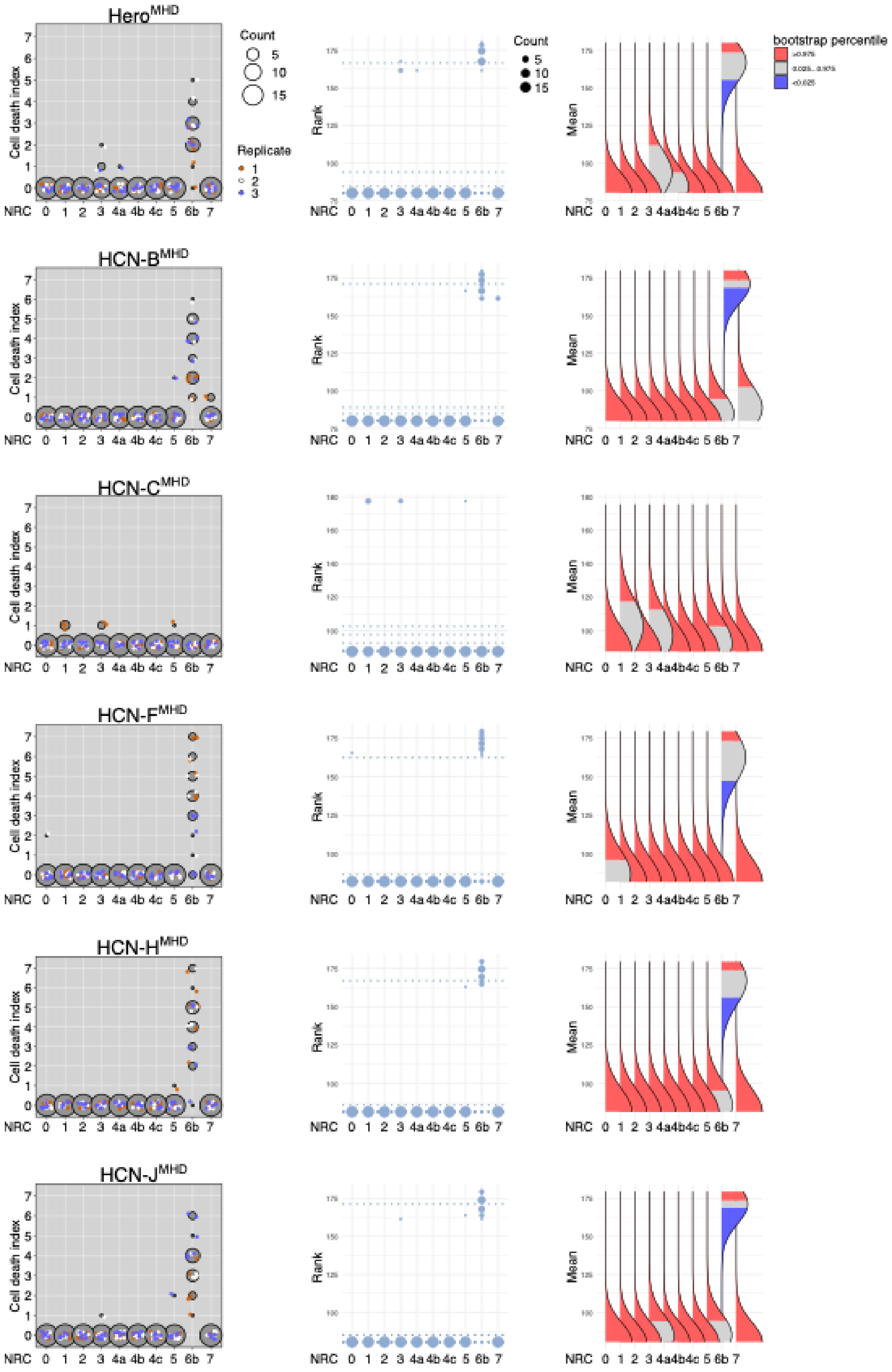
Hero^{MHD}
HCN-B^{MHD}
HCN-C^{MHD}
HCN-F^{MHD}
HCN-H^{MHD}
HCN-J^{MHD}
Cell death index
Rank
Mean
Count
5
10
15
Replicate
1
2
3
bootstrap percentile
NRC 0 1 2 3 4a 4b 4c 5 6b 7

Supplementary Figure S8. Quantification and statistical analysis of cell death upon co-expression of autoactive HCNs and tomato NRCs. (left) Cell death data represented as dots, colored for each biological replicate. The central circle for each cell death category proportionally represents the total number of data points. An asterisk indicates expression of an autoactive NLR MHD mutant (denoted as MHD). Each biological replicate consists of two leaves from three different plants each. **(middle)** Statistical analysis using the besthr R library (MacLean, 2019). The ranked data is shown as dots with their corresponding mean as dashed line. The size of each dot proportionally represents the total number of data points. A bootstrap resampling test, using a lower significance cutoff of 0.025 and an upper cutoff of 0.975 was performed. Mean ranks of test samples falling outside of these cutoffs in the control samples bootstrap population (NRC0) were considered significant. **(right)** The distribution of 1,000 bootstrap sample rank means, blue areas under the curve illustrate the 0.025, and red areas the 0.975 percentiles of the distribution.

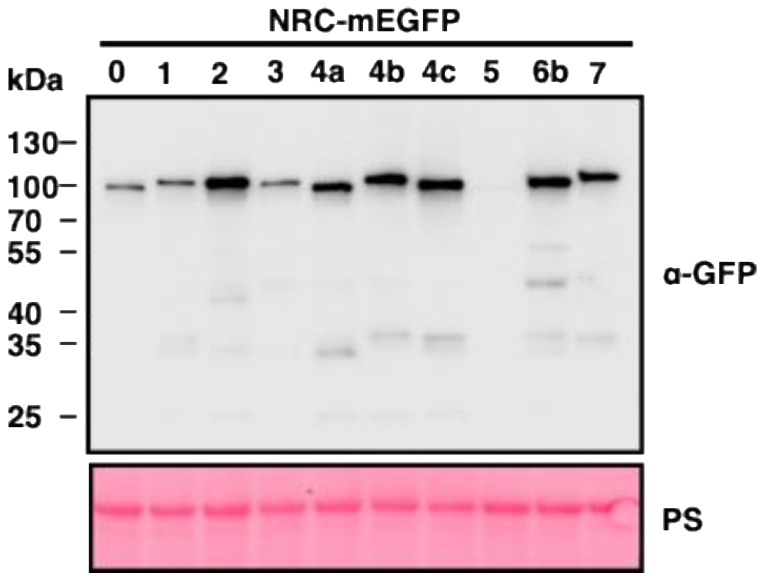

Supplementary Figure S9. Tomato NRC helpers accumulate in planta. Immunoblot analysis of NRC mEGFP accumulation in *N. benthamiana nrc2/3/4* mutant plants at 2 days post-infiltration (dpi) with Agrobacteria transformed with the respective expression constructs, a p19 silencing construct was co-expressed for each infiltration. Ponceau S stain (PS).

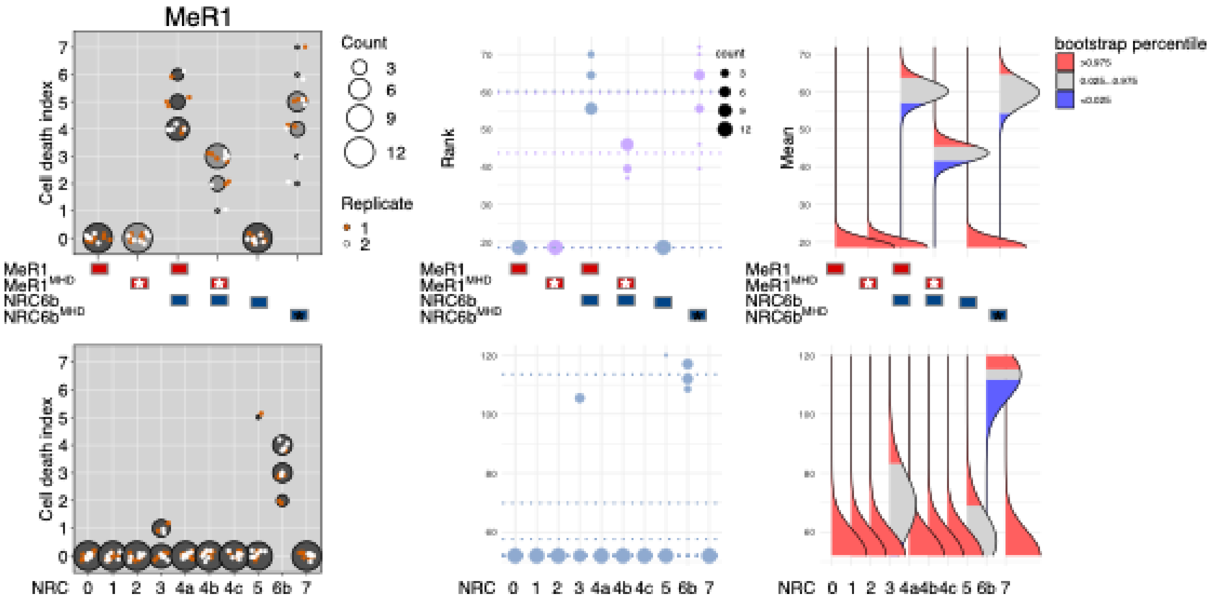

Supplementary Figure S10. Quantification and statistical analysis of cell death upon co-expression of wild-type or autoactive MeR1 and NRC6 or other tomato NRC helpers. (left) Cell death data represented as dots, colored for each biological replicate. The central circle for each cell death category proportionally represents the total number of data points. An asterisk indicates expression of an autoactive NLR MHD mutant (denoted as MHD). Each biological replicate consists of two leaves from three different plants each **(middle)** Statistical analysis using the besthr R library (MacLean, 2019). The ranked data is shown as dots with their corresponding mean as dashed line. The size of each dot proportionally represents the total number of data points. A bootstrap resampling test, using a lower significance cutoff of 0.025 and an upper cutoff of 0.975 was performed. Mean ranks of test samples falling outside of these cutoffs in the control samples bootstrap population (MeR1 alone (top) or NRC0 (bottom)) were considered significant. **(right)** The distribution of 1,000 bootstrap sample rank means, blue areas under the curve illustrate the 0.025, and red areas the 0.975 percentiles of the distribution.

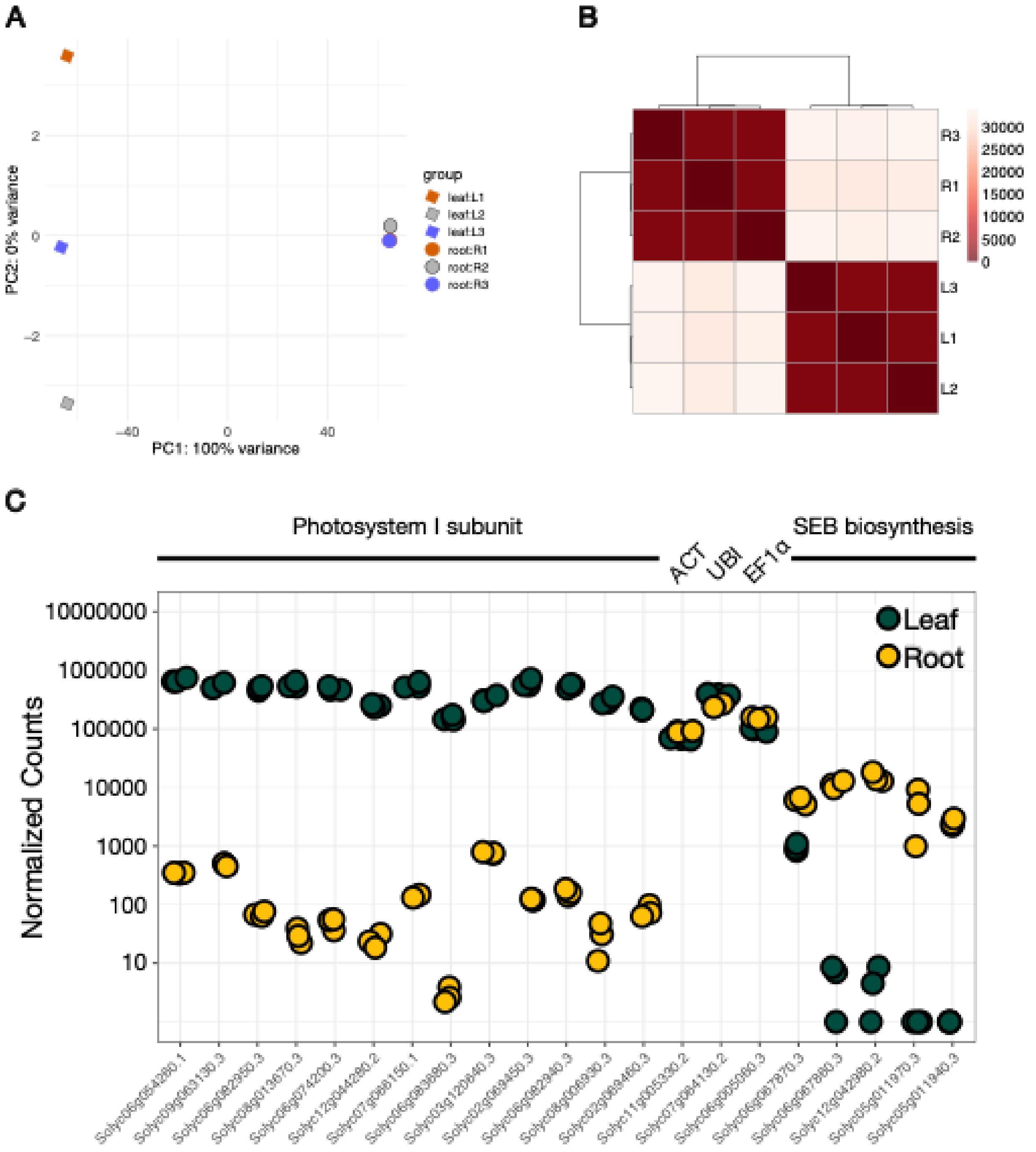

Supplementary Figure S11. The RNA-seq data of leaf and root tissues are consistent across the three biological replicates. (A) Principal component analysis (PCA) after variance stabilizing transformation of the data from the three biological samples from leaves (L1, L2, L3) and roots (R1, R2, R3). Percentages indicate the amount of variation explained by the principal components (PC). **(B)** Heatmap of Poisson distance comparison between each RNA-seq sample using raw counts. The coloring of the scale bar represents the pairwise Poisson distance between RNA-seq samples. **(C)** Normalized counts for the expression of photosystem I subunits, housekeeping genes (Act, UBI, EF1α), and biosynthesis genes of the PCN hatching factor solanoeclepin B (SEB) produced in tomato roots. Normalized counts of all tomato genes and the specific genes shown are available as Supplementary Data 6.

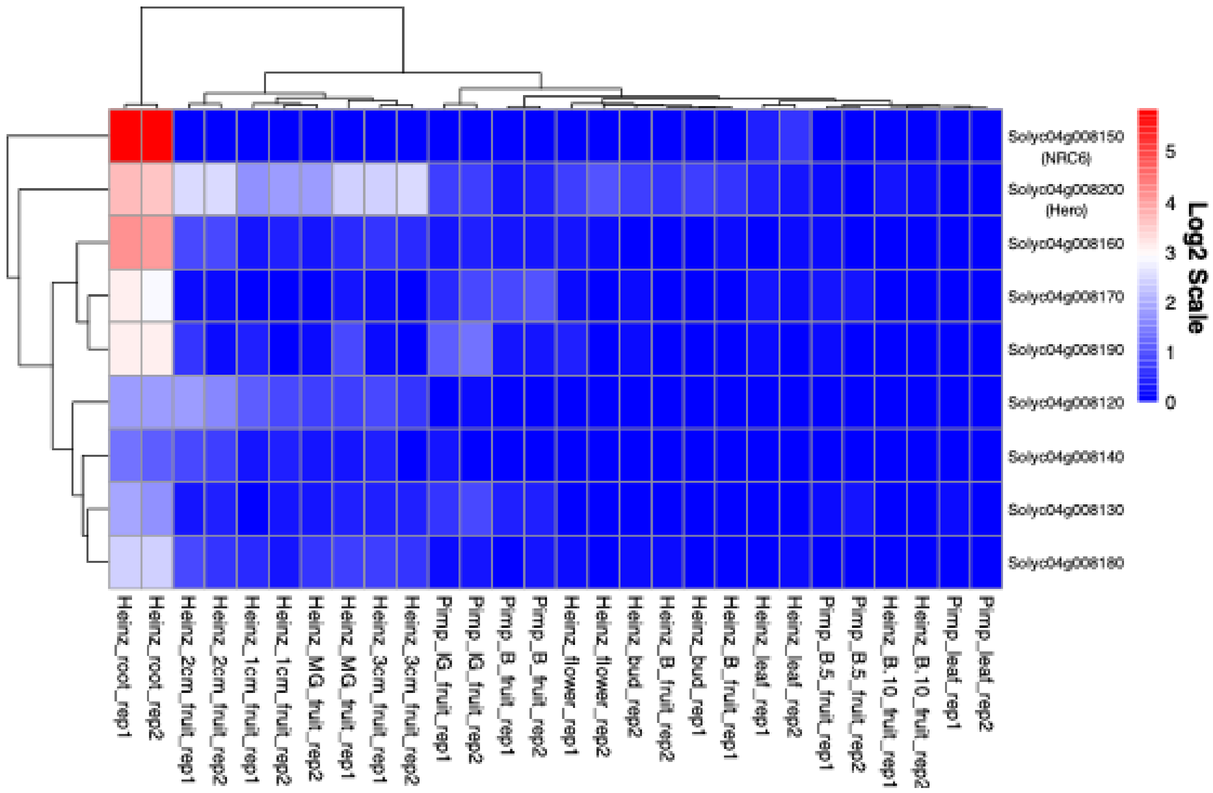

Supplementary Figure S12. Expression profile of NRC6/HCN cluster genes in the Heinz reference tomato line. Heatmap of normalized expression (RPKM) of NRC6/HCN cluster genes based on RNA-seq data (Tomato Genome Consortium 2012) across different tissue types for the Heinz reference genome and *S. pimpinellifolium*.

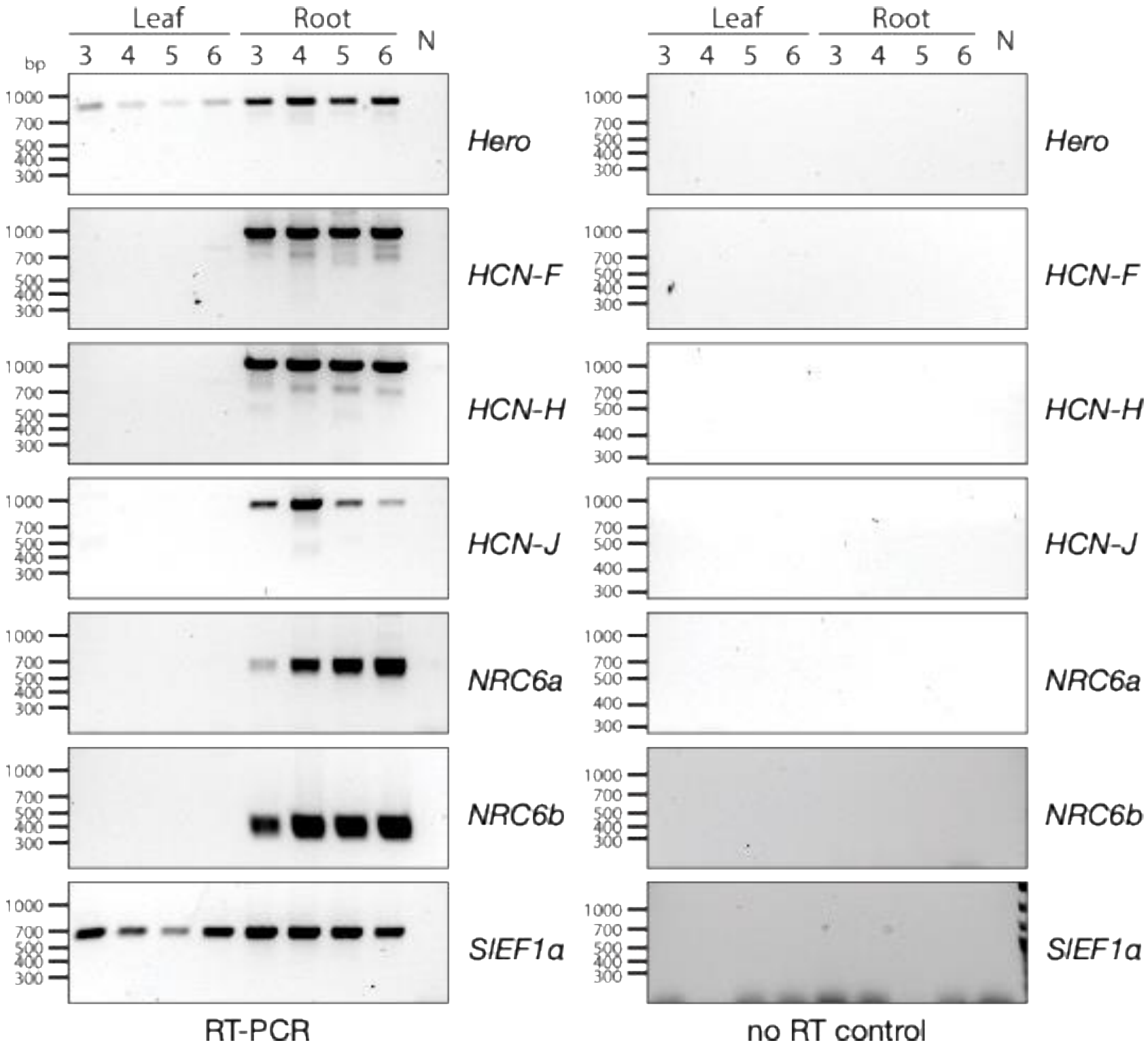

Supplementary Figure S13. HCNs and NRC6 helper genes are nearly exclusively expressed in tomato roots across developmental stages. RT-PCR was performed on cDNA samples generated from leaf and root tissues of three-, four-, five-, or six-week-old plants of the Hero introgression tomato line LA1792 using primers specifically binding to *Hero*, *HCN-F*, *HCN-H*, *HCN-J*, *NRC6a*, or *NRC6b*. The tomato Elongation factor 1α (EF1α) was used as a control. RT-PCRs were run with 25 cycles for EF1α, 31 cycles for *Hero*, and 35 cycles for all other genes as well as for the no RT control.

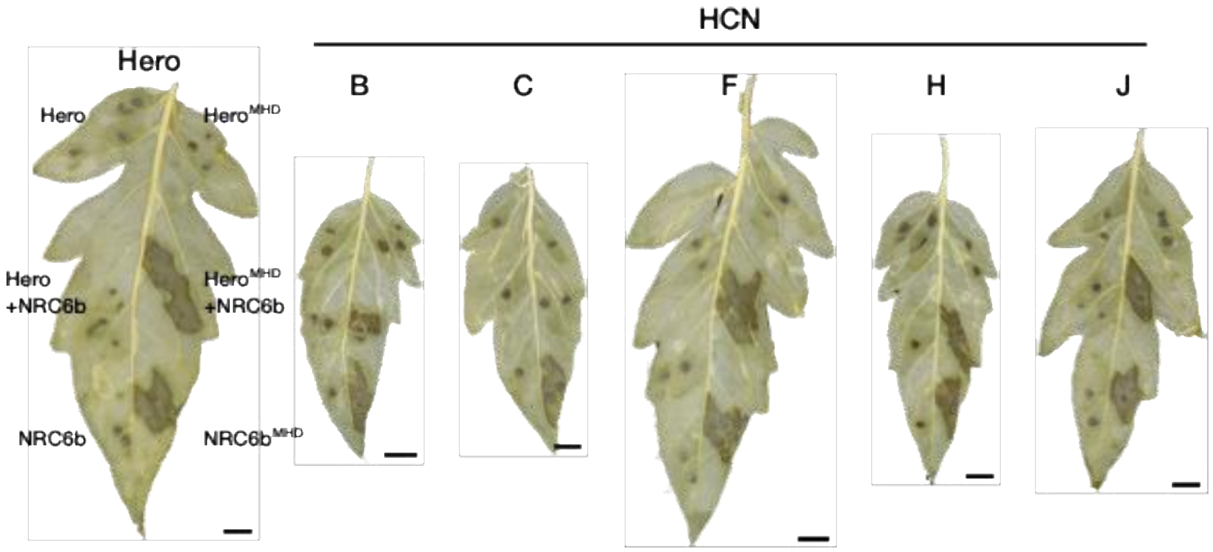

Supplementary Figure S14. HCNs require NRC6b to trigger hypersensitive cell death when transiently expressed in tomato leaves. *R. rhizogenes* strain AS107 carrying NRC6b, or the specified HCN expression constructs were infiltrated into LA1792 tomato plant leaves to express wildtype or autoactive HCNs and the NRC6b helper NRCs (HCN^{MHD} and NRC6b^{MHD}, repecitvely) in the indicated combinations. Leaves with representative cell death phenotypes induced by HCNs when co-expressed with NRC6b were bleached in ethanol and scanned 5-7 days post-infiltration (dpi) with Rhizobium. Images were digitally extracted for comparison; the scale bar represents 1cm. The experiment was repeated three times with similar results. Each biological replicate consists of a leaflet from two different plants each.

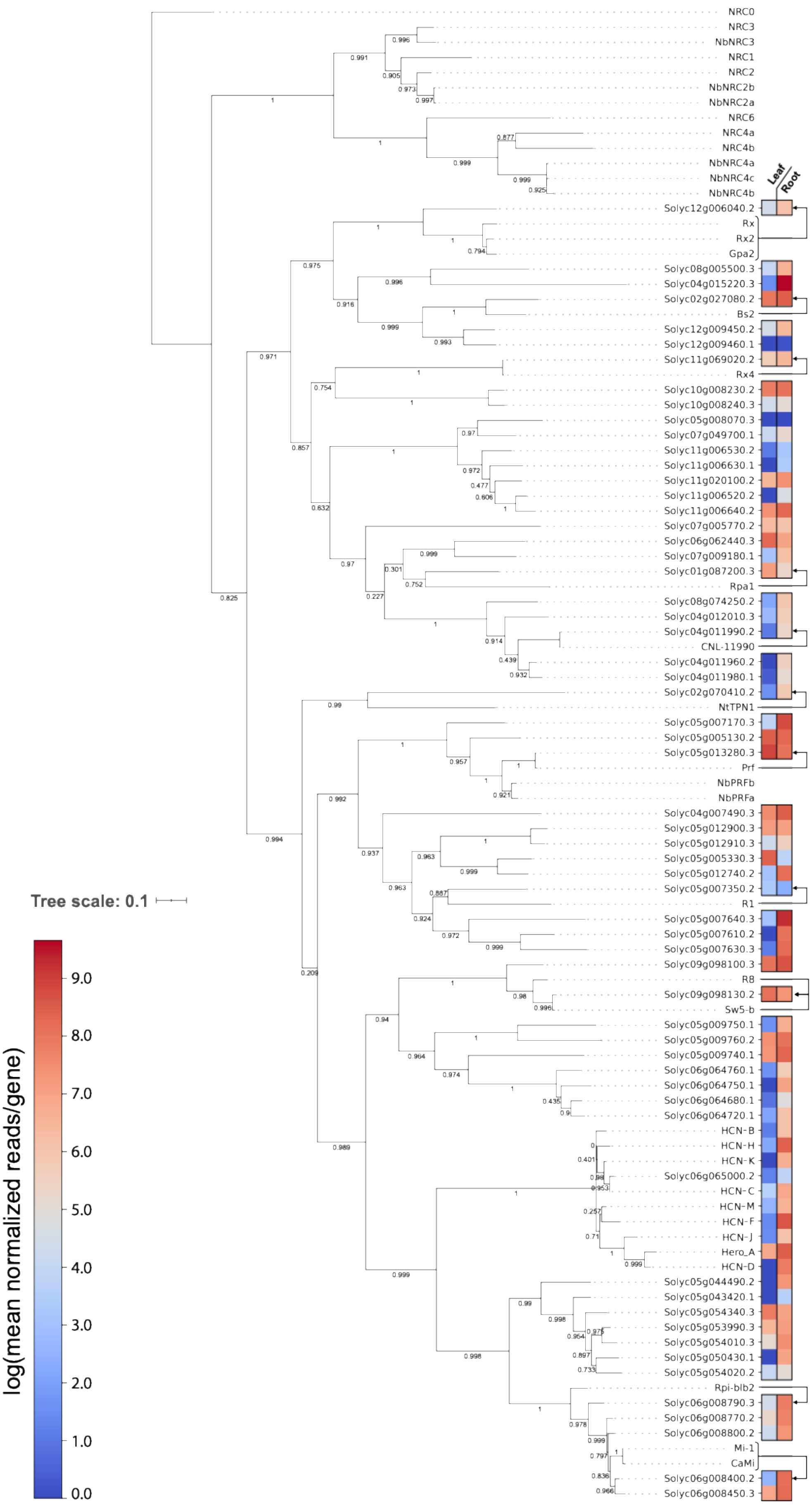
NRC0
NRC3
NbNRC3
NRC1
NRC2
NbNRC2b
NbNRC2a
NRC6
NRC4a
NRC4b
NbNRC4a
NbNRC4c
NbNRC4b
Leaf
Root
Solyc12g006040.2
Rx
Rx2
Gpa2
Solyc08g005500.3
Solyc04g015220.3
Solyc02g027080.2
Bs2
Solyc12g009450.2
Solyc12g009460.1
Solyc11g069020.2
Rx4
Solyc10g008230.2
Solyc10g008240.3
Solyc05g008070.3
Solyc07g049700.1
Solyc11g006530.2
Solyc11g006630.1
Solyc11g020100.2
Solyc11g006520.2
Solyc11g006640.2
Solyc07g005770.2
Solyc06g062440.3
Solyc07g009180.1
Solyc01g087200.3
Rpa1
Solyc08g074250.2
Solyc04g012010.3
Solyc04g011990.2
CNL-11990
Solyc04g011960.2
Solyc04g011980.1
Solyc02g070410.2
NtTPN1
Solyc05g007170.3
Solyc05g005130.2
Solyc05g013280.3
Prf
NbPRFb
NbPRFa
Solyc04g007490.3
Solyc05g012900.3
Solyc05g012910.3
Solyc05g005330.3
Solyc05g012740.2
Solyc05g007350.2
R1
Solyc05g007640.3
Solyc05g007610.2
Solyc05g007630.3
Solyc09g098100.3
R8
Solyc09g098130.2
Sw5-b
Solyc05g009750.1
Solyc05g009760.2
Solyc05g009740.1
Solyc06g064760.1
Solyc06g064750.1
Solyc06g064680.1
Solyc06g064720.1
HCN-B
HCN-H
HCN-K
Solyc06g065000.2
HCN-C
HCN-M
HCN-F
HCN-J
Hero_A
HCN-D
Solyc05g044490.2
Solyc05g043420.1
Solyc05g054340.3
Solyc05g053990.3
Solyc05g054010.3
Solyc05g050430.1
Solyc05g054020.2
Rpi-blb2
Solyc06g008790.3
Solyc06g008770.2
Solyc06g008800.2
Mi-1
CaMi
Solyc06g008400.2
Solyc06g008450.3
Tree scale: 0.1
log(mean normalized reads/gene)
9.0
8.0
7.0
6.0
5.0
4.0
3.0
2.0
1.0
0.0

Supplementary Figure S15. Resistance gene homologs in tomato show varying degrees of expression in roots and leaves. A phylogenetic tree was constructed using selected NRC2/3/4 dependent sensor NLRs from the RefPlant NLR dataset (Kourelis et al. 2021) and the NRC sensor clade of the tomato NLRome. The amino acid sequences of the NB-ARC domain were aligned using MAFFT, and the phylogenetic tree was generated using FastTree. The NRC helper clade served as an outgroup, and the tree was rooted on NRC0. Arrows highlight the closest tomato homolog to the NRC2/3/4 dependent sensor NLRs. The scale bar represents evolutionary distance, measured as substitutions per site. The log-values of means for normalized counts, determined by RNA-seq of two-week-old unchallenged LA1792 tomato plants, were used to indicate the expression of the respective tomato genes in roots and leaves.

Supplementary Table S1. Expression and log2-fold change of HCN and NRC genes in tomato roots and leaves. The expression of genes is given as normalized counts for each independent biological sample of leaves (L1, L2, L3) and roots (R1, R2, R3) of two-week-old unchallenged LA1792 tomato plants, with the corresponding log2-fold change (log2FC) and adjusted P-value (adjP) calculated using DESeq2 with the Wald test and Benjamini-Hochberg correction for multiple testing.. Expression data and log2-fold changes for all tomato genes is shown in Supplementary Data 5 and 6.

Gene	L1	L2	L3	R1	R2	R3	log2FC	adjP
Hero	1046.31	1032.22	762.56	4729.93	4810.30	4365.15	2.29	7.32E-61
HCN-B	6.53	0.00	0.00	452.44	464.87	435.89	7.66	4.68E-23
HCN-C	26.15	36.76	50.43	947.75	997.42	969.90	4.71	3.52E-79
HCN-D	0.00	0.00	0.00	2452.05	3337.51	2098.27	13.04	5.30E-27
HCN-F	3.26	5.88	0.00	5528.69	5588.93	5158.00	10.66	2.20E-70
HCN-H	0.00	0.00	24.20	4639.44	3896.83	4546.73	9.10	4.38E-06
HCN-J	0.00	5.88	4.03	406.18	434.96	491.72	7.05	3.07E-27
HCN-K	0.00	0.00	0.00	854.54	734.55	726.86	11.28	1.43E-20
HCN-M	9.80	4.41	24.20	621.17	746.10	788.89	5.90	6.27E-45
HCN-N	0.00	0.00	0.00	83.00	104.93	113.34	8.33	2.50E-10
NRC0	31.06	54.40	0.00	48.30	90.77	47.36	1.12	0.34
NRCX	214.16	282.31	292.51	6381.87	6936.32	6442.56	4.65	2.57E-293
NRC1	2516.05	2371.75	2178.76	1598.87	1568.28	1672.53	-0.55	3.07E-08
NRC2	4195.06	3559.83	2785.98	8738.68	8874.51	8966.58	1.33	3.44E-20
NRC3	3655.56	3923.03	4502.77	5900.17	6118.35	5227.93	0.51	7.35E-06
NRC4a	4172.17	3631.89	3788.62	16352.71	14693.26	14601.09	1.98	2.74E-112
NRC4b	3264.82	3333.40	3324.63	24577.03	20867.75	21654.92	2.76	3.66E-236
NRC4c	0.00	0.00	0.00	86660.85	75688.36	81911.12	18.00	1.25E-51
NRC5	14.71	14.70	0.00	31201.80	23106.58	25870.65	11.30	2.28E-216
NRC6a	0.00	0.00	0.00	8852.98	5469.31	6324.71	11.39	7.40E-67
NRC6b	8.17	0.00	0.00	8243.37	7128.36	7111.35	14.43	6.21E-33
NRC7	480.65	367.60	484.17	30304.39	28525.00	28782.07	6.05	0.00
